# Enhancing preoperative HER2 status classification of invasive breast cancers using machine learning models based on clinicopathological and MRI features: a multicenter study

**DOI:** 10.3389/fcell.2025.1669651

**Published:** 2025-11-26

**Authors:** Suhong Zhao, Zhaohua Li, Yanan Wang, Fang Zhao, Peipei Chen, Guodong Pang

**Affiliations:** 1 Department of Radiology, The Second Hospital of Shandong University, Jinan, China; 2 Department of Radiology, Linglong Yingcheng Hospital, Yantai, China; 3 Department of Radiology, Qilu Hospital of Shandong University, Jinan, China

**Keywords:** magnetic resonance imaging, clinicopathological features, breast cancer, humanepidermal growth factor 2, machine learning

## Abstract

**Rationale and Objectives:**

The human epidermal growth factor receptor 2 (HER2) gene status is crucial for determining treatment efficacy. This study assessed preoperative HER2 classification in breast cancer using machine learning based on clinicopathological and MRI characteristics.

**Materials and Methods:**

This retrospective study involved 1,015 patients (1,030 lesions) across two centers. Patients were divided into training, internal validation, and external validation sets. Nomograms were developed using clinicopathological and MRI features. Predictive models were constructed using decision trees (DT), support vector machines (SVM), k-nearest neighbors (k-NN), artificial neural networks (ANN), and multivariable logistic regression (LR). Model performance was evaluated using receiver operating characteristic curves, decision curve analysis, and calibration curves. Model interpretability was achieved by developing nomograms and employing SHAP (SHapley Additive exPlanations) analysis.

**Results:**

Key variables for distinguishing HER2-positive from HER2-negative cases included regional N category, estrogen receptor, PR (progesterone receptor) status, Ki-67 status, lesion number, distribution quadrant, and accompanying signs. The SVM model achieved the highest AUC of 0.86 (95% confidence interval (CI): 0.81–0.90) in the training set, while the ANN model had an AUC of 0.77 (95% CI: 0.67–0.86) in the internal validation set. In the external validation set, the LR model achieved the highest AUC of 0.66 (95% CI: 0.56–0.76), although the overall performance was modest. For HER2-low versus HER2-zero differentiation, Ki-67 status, lesion number, distribution quadrant, mass shape, early enhancement rate, and ADC (apparent diffusion coefficient) were significant. The SVM model attained the highest AUC of 0.87 (95% CI: 0.83–0.91) in the training set, while the LR model demonstrated superior generalizability, yielding the highest AUCs in both the internal and external validation sets (internal: 0.67, 95% CI: 0.58–0.76; external: 0.74, 95% CI: 0.65–0.83). Radiologists benefited from the nomogram for improved diagnostic accuracy, especially junior radiologists. SHAP analysis revealed that PR status was paramount for HER2-positive classification, whereas mass shape and ADC values were dominant for identifying HER2-low status.

**Conclusion:**

Integrating machine learning with clinicopathological and MRI characteristics improves the accuracy of HER2 status classification in breast cancer and enhances diagnostic capabilities for radiologists in clinical practice.

## 1 Introduction

Breast cancer is a highly heterogeneous disease with complex clinical and pathological manifestations (Brenner et al., 2020; Szymiczek et al., 2021). The treatment of breast cancer depends on the TNM stage and pathological characteristics, particularly the molecular subtype (Barzaman et al., 2020). The human epidermal growth factor receptor 2 (HER2) gene plays a crucial role in breast cancer, as it not only determines the molecular subtype but also directly influences treatment selection and efficacy (Barzaman et al., 2020). Traditionally, HER2 status categorizes tumors as HER2-positive or HER2-negative (Wolff et al., 2018). HER2-targeted therapies are effective in HER2-positive patients (Loiblm et al., 2022). However, the American Society of Clinical Oncology and American Pathological Society guidelines have redefined HER2-negative cases into two subcategories: HER2-low expression and HER2-zero expression (Tarantino et al., 2023). HER2-low breast cancer patients account for more than half of the traditional HER2-negative cohort (Marchio et al., 2021). Compared to HER2-positive and HER2-zero expression breast cancer, the prevalence of estrogen receptors (ER) and progesterone receptors (PR) is higher in HER2-low tumors, while Ki67 levels tend to be lower (Marchio et al., 2021; Zhang et al., 2022). HER2-low breast cancer may benefit from new therapeutic interventions, such as antibody-drug conjugates (Marchio et al., 2021; Modi et al., 2022; Xin et al., 2022; Zhang et al., 2022; Yang et al., 2023). Therefore, accurately determining HER2 expression status in breast cancer patients is crucial for identifying potential candidates for anti-HER2 therapies. Clinically, HER2 status is primarily assessed through biopsy; however, this invasive procedure may introduce errors due to tumor heterogeneity and variability in specimen quantity and quality (Chen et al., 2023). Thus, it is essential to develop non-invasive methods for predicting HER2 status.

Multiparameter breast magnetic resonance imaging (MRI) techniques, including T2-weighted imaging (T2WI), T1-weighted dynamic contrast-enhanced MR imaging (DCE-MRI), and diffusion-weighted imaging (DWI), non-invasively reflect tumor vascularity and cellular density, providing comprehensive lesion characterization (Yang et al., 2017; Xie et al., 2019; Xu et al., 2022). Artificial intelligence (AI) models, particularly radiomics, have been applied to breast MRI for tasks like benign-malignant differentiation and HER2 status prediction (Chen et al., 2020; Kim et al., 2021; Bian et al., 2023; Ramtohul et al., 2023; Zhang et al., 2023; Chen et al., 2024; Guo et al., 2024; Peng et al., 2024; Zheng et al., 2024). However, radiomics models often suffer from poor interpretability and require specialized software for feature extraction, limiting their clinical adoption. In contrast, using routinely available clinicopathological data and qualitative MRI features assessed by radiologists offers a more transparent and potentially more accessible approach. Zhou et al. (2025) recently used BI-RADS features with machine learning (ML) algorithms to classify HER2 status, suggesting its promise, but their study lacked external validation. Other approaches using synthetic MRI or DWI have been limited by small sample sizes (Zhan et al., 2024).

Therefore, this study aims to develop and validate a clinically practical framework for preoperative HER2 status classification by integrating readily available clinicopathological characteristics and conventional MRI features using ML. Our approach distinctively focuses on using features that are directly interpretable by clinicians, thereby enhancing the model’s transparency and potential for integration into routine workflows. We constructed predictive models and clinical nomograms to first differentiate HER2-positive from HER2-negative breast cancer, and then to distinguish the clinically critical HER2-low from HER2-zero subcategories within the HER2-negative group. Furthermore, we conducted an external validation to assess generalizability and evaluated whether the nomograms could enhance the diagnostic performance of radiologists with varying experience levels. This research may contribute to more precise, non-invasive HER2 stratification to guide clinical decision-making.

## 2 Materials and Methods

### 2.1 Ethics statement

The study conformed to the provisions of the Declaration of Helsinki and was approved by the Ethics Committee of the Second Hospital of Shandong University (Approval No: KYLL2025281; Date: 21 Feb 2025). Informed consent was waived for all patients due to the nature of the retrospective analysis.

### 2.2 Patients

This retrospective study involved two centers: the Second Hospital of Shandong University (referred to as “Center 1”) and the Qilu Hospital of Shandong University (referred to as “Center 2”). Consecutive breast cancer patients who underwent MRI at Center 1 (July 2020 to December 2023) and Center 2 (January 2023 to August 2023) were included. For the selection of lesions, bilateral breast lesions were measured separately, and among multiple lesions on the same side, the largest lesion was selected for measurement. Inclusion criteria: 1) breast cancer patients with HER2-positive, HER2-low, or HER2-zero expression confirmed by pathology, with complete clinicopathological data and no distant metastasis; 2) patients who underwent multiparameter MRI examination, including T2WI, DWI, and DCE-MRI. Exclusion criteria: 1) patients with incomplete clinicopathological data or distant metastasis; 2) patients with non-invasive breast cancer; 3) patients who underwent radiotherapy, chemotherapy, or breast biopsy before MRI examination; 4) patients with incomplete MRI image data or poor image quality; 5) patients exhibiting pure non-mass enhancement (NME) on DCE-MRI.

### 2.3 MRI examination

At Center 1, MRI examinations were performed using a 3.0T MRI imaging system (GE Discovery MR750, United States) with an 8-channel dedicated breast surface coil. The patient was positioned in the prone position, and both breasts were scanned simultaneously. The examination was conducted 7–14 days after menstruation. The scanning included T2 IDEAL, T1-weighted fast spin echo, DWI, and DCE-MRI sequences. The single-shot echo-planar imaging technology for transverse plane scanning was used in DWI. The b-values were set at 0 s/mm^2^ and 800 s/mm^2^, and the scanning parameters were as follows: repetition time (TR): 3,000 ms, echo time (TE): 49.5 ms, slice thickness: 5 mm, slice gap: 1.0 mm, field of view (FOV): 360 × 360 mm, matrix: 128 × 96, and number of excitations (NEX): 4. In DCE-MRI scanning, the Vibrant-Flex technology was used. Following the plain scan, the contrast agent gadodiamide (0.2 mmol/kg body weight) was injected at a rate of 2 mL/s, followed by a 20 mL saline flush. A mask scan was conducted before the administration of the contrast agent, and dynamic enhancement images were acquired immediately after the saline injection. A total of 7 sequences were obtained without intervals, each lasting 60 s. The parameters for these scans were as follows: TR: 3.9 ms, TE: 1.7 ms, flip angle: 5°, FOV: 360 × 360 mm, matrix: 348 × 348, slice thickness: 1.8 mm, and NEX: 0.7.

At Center 2, breast MRI scans were also performed using a 3.0T MRI system (GE SIGNA Architect 3.0T, United States) and an 8-channel dedicated breast surface coil. Patients were positioned in the prone position. The breast MRI protocol included several sequences: an axial pre-contrast 2D fast spin echo T2-weighted fat-suppressed sequence (TR: 4,000–6,000 ms, TE: 80–100 ms, matrix: 320 × 256, slice thickness: 3–4 mm, FOV: 36 × 36 cm, NEX: 2, and scan time: 120–180 s); an axial pre-contrast diffusion-weighted echo-planar imaging sequence (TR: 5,000–8,000 ms, TE: 60–80 ms, matrix: 128 × 128, slice thickness: 4–5 mm, FOV: 360 mm × 360 mm, NEX: 2–4, and scan time: 180–240 s); and an axial dynamic 3D spoiled gradient-echo T1-weighted fat-suppressed sequence (flip angle: 10°–12°, TR: 4–6 ms, TE: 1.5–2.5 ms, matrix: 512 × 512, slice thickness: 1–2 mm, FOV: 360 mm × 360 mm, and NEX: 1). Additionally, a sagittal 3D spoiled gradient-echo post-contrast T1-weighted sequence was performed. The DCE-MRI utilized axial imaging and included one pre-contrast and 5 post-contrast dynamic series. Contrast-enhanced axial images were captured at 1.5, 3, 4.5, and 6 min post-contrast injection, followed by a delayed sagittal image obtained 8 min after injection. A bolus of 0.1 mmol/kg of gadodiamide contrast agent was administered at a rate of 2 mL/s, accompanied by a 20 mL saline flush.

### 2.4 Image analysis

All MRI images were evaluated on the GE AW4.6 workstation by two radiologists with 8 and 26 years of experience, respectively, utilizing the Breast Imaging Reporting and Data System (BI-RADS) (Pinker et al., 2013). Both radiologists were fully blinded to all patient information, including clinical history, laboratory results, and pathological findings (e.g., HER2, ER, PR, and Ki-67 status). In cases of initial disagreement, a consensus decision was reached through a joint re-review of the images and direct discussion between the two radiologists. For early enhancement rate (EER) and time-signal intensity curve (TIC) evaluation, the region of interest (ROI) was analyzed using the T1 Perfusion software. During ROI delineation, areas exhibiting liquefied necrosis and hemorrhage were avoided. The ROI was defined in the most apparent and prominent area of enhancement and was smaller than the overall size of the lesion. The EER of TIC was calculated using the formula (SI_post - SI_pre)/SI_pre × 100%, where SI represents signal intensity. Here, SI_pre and SI_post denote the signal intensities before and after enhancement, respectively. The signal intensity measured 2 min after enhancement, or the highest peak signal intensity within 2 min, was referred to as SI_post. Delayed enhancement in TIC indicated enhancement occurring either 2 min post-enhancement or when the curve changes. TIC was categorized into three distinct types: progressive type (Type I, characterized by a consistent increase in signal intensity over time), platform type (Type II, where signal intensity remains constant after the initial enhancement), and washout type (Type III, in which signal intensity decreases after reaching peak enhancement). For apparent diffusion coefficient (ADC) measurement, a small ROI was selected in the darkest part of the lesion on the ADC map, while avoiding areas of liquefied necrosis, significant noise, or unenhanced regions (Baltzer et al., 2020). Multiple measurements were taken, ensuring consistent ROI size, and the average minimum ADC value for each lesion was recorded.

### 2.5 Assessment of histologic grade and tumor biomarkers in breast cancer

The histologic grade of tumor tissue was assessed using the Bloom-Richardson method (29), which evaluates tubular formation, nuclear pleomorphism, and mitotic count, each of which was assigned a score of 1, 2, or 3 points. Total scores of 3–5, 6–7, and 8–9 were classified as histological grades I, II, and III, respectively (Rakha et al., 2008). HER2 status was determined using immunohistochemistry (IHC) and *in situ* hybridization (ISH). HER2 status was categorized into HER2-positive (IHC score 3+ or IHC score 2+ with positive ISH amplification) and HER2-negative (IHC score 0 or 1+, or IHC score 2+ without ISH amplification) (Wolff et al., 2018; Tarantino et al., 2023). HER2-negative status was further classified into HER2-low (IHC score 1+ or IHC score 2+ without ISH amplification) and HER2-zero (IHC score 0). The criteria for evaluating ER and PR results were as follows: a positive percentage of ≥1% was considered positive, while a percentage of <1% was deemed negative. The hormone receptor (HR) status was considered positive if either ER or PR, or both, were positive (Allison et al., 2020). If both ER and PR were negative, HR was classified as negative. The Ki-67 index was measured based on the positive staining area, with ≥20% considered positive and <20% considered negative (Lee et al., 2023). Based on the ER, PR, HER2 status, and Ki-67 index, breast cancer was categorized into four molecular subtypes (Lawton, 2023): Luminal A (ER+, PR+, HER2-negative, and low Ki-67), Luminal B (ER+, PR-negative or low, HER2-negative, and high Ki-67), HER2-positive (ER-negative, PR-negative, and HER2-positive), and triple-negative (ER-negative, PR-negative, and HER2-negative).

### 2.6 Collection of clinicopathologic and MRI features

The clinical characteristics examined included patient age, menopausal status, regional N category, and primary T category. The pathological characteristics consisted of histological grade, HR status, ER status, PR status, Ki-67 status, and molecular subtype. Furthermore, a range of MRI features was observed and analyzed, including fibroglandular tissue component, background parenchymal enhancement, tumor diameter, tumor distribution, lesion location, distribution quadrant of lesions, signal on T2WI, intratumoral edema, peritumoral edema, lesion enhancement type, mass shape, mass margin, EER, TIC, ADC, increased vascularity or adjacent vessel sign, lymphadenectasis (axillary or internal mammary), and accompanying signs (such as nipple inversion, skin retraction, and pectoralis muscle invasion), and BI-RADS classifications.

### 2.7 Construction of ML predictive models

LASSO regression was used to select variables with non-zero coefficients for data dimensionality reduction and feature screening. Subsequently, a multivariable logistic regression model was constructed using the selected variables. Variables with p-values less than 0.1 in the multivariable logistic regression underwent further evaluation through stepwise regression for refined variable selection. Ultimately, the variables with p-values less than 0.05 in the stepwise regression were used for the construction of the final predictive models and the nomogram model. These selected variables were also used to train predictive models on the training set via five-fold cross-validation employing 5 ML algorithms: decision trees (DT), support vector machines (SVM), k-nearest neighbors (KNN), artificial neural networks (ANN), and multivariable logistic regression (LR). Each model was then applied to both the internal and external validation sets. The predictive performance of each model was assessed using receiver operating characteristic (ROC) curves. The area under the curve (AUC), sensitivity, and specificity were calculated. A calibration curve was used to visualize the degree of calibration of the predictive models. Furthermore, the clinical applicability of the models was evaluated through decision curve analysis (DCA). To enhance model interpretability, Shapley Additive exPlanations (SHAP) analysis was conducted to quantify the contribution of individual clinical, pathological, and imaging features to the model’s predictions.

### 2.8 Validation of predictive model by independent radiological assessment

To further validate the predictive value of the ML models, two additional radiologists with varying levels of seniority (Radiologist 1 with 18 years of experience and Radiologist 2 with 6 years of experience) were invited to assess the images of each patient in the external validation set. Both radiologists were blinded to pathological information. They classified each lesion as representing HER2-positive, HER2-low, or HER2-negative breast cancer. Each radiologist was required to reclassify the images with the assistance of the optimal model, which exhibited the highest AUC, to investigate the incremental benefit of the model for diagnostic radiologists.

### 2.9 Statistical analysis

Categorical variables are presented as counts and percentages and were compared with chi-square tests. Continuous variables are expressed as means and standard deviations or as median (interquartile range). The Kolmogorov-Smirnov test was applied to determine the normality of the distribution. If a variable was normally distributed, a t-test was utilized to compare group differences; if not, the Wilcoxon test was used to evaluate significant differences in medians between groups. To control the risk of false positives arising from multiple comparisons, the False Discovery Rate (FDR) correction was applied to the p-values from all univariate comparisons. Features with an FDR-adjusted p-value of ≤0.05 were considered statistically significant. The inter-observer agreement for MRI features was assessed using intraclass correlation coefficients (ICCs) for continuous variables and kappa coefficients for categorical variables. ICC values were classified as follows: less than 0.5, poor; between 0.5 and 0.75, moderate; between 0.75 and 0.9, good; greater than 0.90, excellent. Kappa coefficients were classified as follows: less than 0.00, poor; between 0.00 and 0.20, slight; between 0.21 and 0.40, fair; between 0.41 and 0.60, moderate; between 0.61 and 0.80, substantial; between 0.81 and 1.00, almost perfect. All statistical analyses were performed using R software (version 4.4.2).

## 3 Results

### 3.1 Clinicopathological characteristics of HER2-positive, HER2-negative, HER2-low and HER2-zero breast cancer patients

**FIGURE 1:**
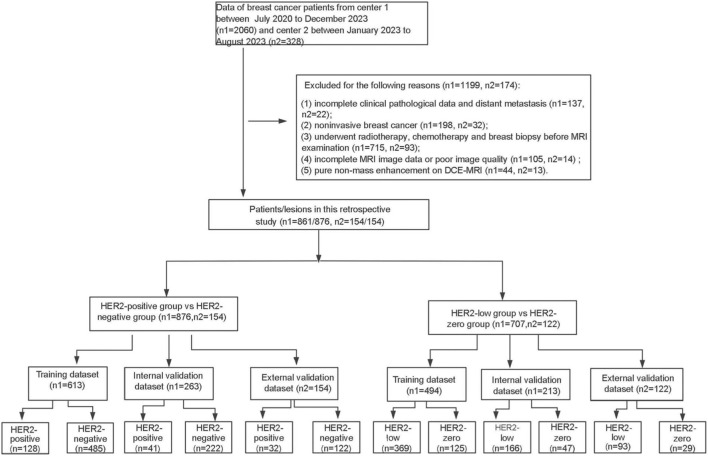
The study flow chart of patient enrollment.

The flow chart of this study is illustrated in Figure 1. Consecutive breast cancer patients who underwent MRI at Center 1 (July 2020 to December 2023) and Center 2 (January 2023 to August 2023) were initially screened. A total of 57 patients exhibiting pure NME on DCE-MRI were excluded based on the predefined exclusion criterion, as the standard BI-RADS features for masses are not directly applicable to this enhancement type. After applying all other inclusion and exclusion criteria, a total of 861 patients (including 15 bilateral patients) with 876 lesions from Center 1 and 154 patients with 154 lesions from Center 2 were finally included. The patient lesions from Center 1 were allocated to the training set and internal validation set in a 7:3 ratio, while those from Center 2 served as the external validation set.

For patients with HER2-positive and HER2-negative breast cancers, the training set consisted of 613 lesions (128 HER2-positive and 485 HER2-negative), the internal validation set comprised 263 lesions (41 HER2-positive and 222 HER2-negative), and the external validation set included 154 lesions (Figure 1). The clinicopathological characteristics of these HER2-positive and HER2-negative breast cancer patients are shown in Table 1. After FDR correction, histological grade, primary T category, HR status, ER status, PR status, Ki-67 status, and molecular subtype were statistically significant between HER2-positive and HER2-negative breast cancer patients in the training set (FDR-adjusted p-values ≤0.05). Compared with HER2-negative patients, HER2-positive patients were more likely to have a higher histological grade, negative ER, negative PR, and higher Ki-67 status. In both the internal and external validation sets, no differences were found in histological grade and tumor staging (FDR-adjusted p-values >0.05), while ER status and Ki-67 status were significantly different (FDR-adjusted p-values ≤0.05). Additionally, there were no statistical differences in patient age, menopausal status, or lymph node staging across the training, internal validation, and external validation sets (FDR-adjusted p-values >0.05).

**TABLE 1:** Clinicopathological and MRI features of HER2-positive and HER2-negative breast cancer patients in the training, internal validation, and external validation sets.

Features		Training set (n = 613 lesions)		FDR-adjusted p-value	Internal validation set (n = 263 lesions)		FDR-adjusted p-value	External validation set (n = 154 lesions)		FDR-adjusted p-value
		HER2-positive (n = 128 lesions)	HER2-negative (n = 485 lesions)		HER2-positive (n = 41 lesions)	HER2-negative (n = 222 lesions)		HER2- positive (n = 32 lesions)	HER2- negative (n = 122 lesions)	
Age (Mean ± SD)		53.64 ± 10.99	52.66 ± 10.0	0.46	53.08 ± 11.96	52.15 ± 9.77	0.88	54.59 ± 9.91	52.19 ± 10.08	0.41
Menopausal status	Postmenopausal	74, 57.8%	267, 55.1%	0.69	21, 51.2%	115, 51.8%	1.00	18, 56.2%	82, 67.2%	0.57
	Premenopausal	54, 42.2%	218, 44.9%		20, 48.8%	107, 48.2%		14, 43.8%	40, 32.8%	
Tumor diameter (cm)		2.45 (1.9, 3)	2.1 (1.6, 2.9)	**0.02**	2.2 (1.7, 2.9)	2.1 (1.6, 2.8)	0.63	2.45 (1.9, 3.4)	2 (1.5, 2.58)	0.07
Histological grade	I	3, 2.34%	31, 6.4%	**0.05**	2, 4.9%	14, 6.3%	0.98	0, 0%	8, 6.6%	0.41
	II	73, 57.03%	310, 63.9%		24, 58.5%	143, 64.4%		18, 56.2%	76, 62.3%	
	III	52, 40.63%	144, 29.7%		15, 36.6%	65, 29.3%		14, 43.8%	38, 31.1%	
Regional N category	N0	79, 61.72%	282, 58.15%	0.51	27, 65.9%	134, 60.4%	0.84	18, 56.3%	73, 59.84%	1.00
	N1	46, 35.94%	175, 36.08%		13, 31.7%	81, 36.5%		13, 40.6%	42, 34.42%	
	N2	3, 2.34%	21, 4.33%		1, 2.4%	2, 0.9%		1, 3.1%	6, 4.92%	
	N3	0, 0%	7, 1.44%		0, 0%	5, 2.2%		0, 0%	1, 0.82%	
Primary T category	T1	42, 32.8%	223, 46%	**0.02**	18, 43.9%	104, 46.8%	1.00	10, 31.3%	65, 53.3%	0.23
	T2	82, 64.1%	238, 49.1%		21, 51.2%	107, 48.2%		20, 62.5%	50, 41%	
	T3	4, 3.1%	24, 4.9%		2, 4.9%	11, 5%		2, 6.2%	7, 5.7%	
HR status	Positive (ER and/or PR positive)	67, 52.3%	401, 82.7%	**<0.001**	23, 56.1%	179, 80.6%	**0.01**	16, 50%	91, 74.6%	0.07
	Negative (ER and PR negative)	61, 47.7%	84, 17.3%		18, 43.9%	43, 19.4%		16, 50%	31, 25.4%	
ER status	Positive≥1%	59, 46.1%	389, 80.2%	**<0.001**	19, 46.3%	179, 80.6%	**<0.001**	14, 43.8%	89, 73%	**0.05**
	Negative<1%	69, 53.9%	96, 19.8%		22, 53.7%	43, 19.4%		18, 56.2%	33, 27%	
PR status	Positive≥1%	50, 39.1%	361, 74.4%	**<0.001**	18, 43.9%	162, 73%	**0.01**	15, 46.9%	83, 68%	0.17
	Negative<1%	78, 60.9%	124, 25.6%		23, 56.1%	60, 27%		17, 53.1%	39, 32%	
Ki-67 status	Low (<20%)	14, 10.9%	165, 34%	**<0.001**	5, 12.2%	72, 32.4%	**0.05**	4, 12.5%	46, 37.7%	**0.05**
	High (≥20%)	114, 89.1%	320, 66%		36, 87.8%	150, 67.6%		28, 87.5%	76, 62.3%	
Molecular subtype	Luminal A	0	167, 34.43%	**<0.001**	0	68, 30.63%	**<0.001**	0	41, 33.61%	**<0.001**
	Luminal B	0	241, 49.69%		0	112, 50.45%		0	51, 41.80%	
	HER2- enriched	128	0, 0		41	0, 0		32	0, 0	
	Triple-negative	0	77, 15.88%		0	42, 18.92%		0	30, 24.59%	
Histological subtype	IDC	99, 77.3%	375, 77.32%	0.59	35, 85.4%	163, 73.4%	0.84	20, 62.5%	70, 57.4%	1.00
	IDC + DCIS	20, 15.6%	60, 12.37%		5, 12.2%	27, 12.2%		9, 28.1%	41, 33.6%	
	ILC	2, 1.6%	18, 3.71%		0, 0%	8, 3.6%		1, 3.1%	3, 2.5%	
	Mucinous	0, 0%	7, 1.44%		0, 0%	5, 2.2%		2, 6.3%	8, 6.5%	
	Other types	7, 5.5%	25, 5.16%		1, 2.4%	19, 8.6%		0, 0%	0, 0%	
FGT	Fatty or scattered	18, 14.1%	54, 11.1%	0.58	5, 12.2%	34, 15.3%	0.98	3, 9.4%	14, 11.5%	1.00
	Heterogeneous or extreme	110, 85.9%	431, 88.9%		36, 87.8%	188, 84.7%		29, 90.6%	108, 88.5%	
BPE	Minimal or mild	114, 89.1%	421, 86.8%	0.67	32, 78%	198, 89.2%	0.28	28, 87.5%	111, 91%	0.73
	Moderate or marked	14, 10.9%	64, 13.2%		9, 22%	24, 10.8%		4, 12.5%	11, 9%	
Lesion number	Single lesion	59, 46.1%	339, 69.9%	**<0.001**	18, 43.9%	155, 69.8%	**0.02**	14, 43.8%	82, 67.2%	0.72
	Multifocal or multicentric lesions	69, 53.9%	146, 30.1%		23, 56.1%	67, 30.2%		18, 56.2%	40, 32.8%	
Lesion location	Left	57, 44.5%	238, 49.1%	0.36	18, 43.9%	118, 53.2%	0.63	19, 59.4%	70, 57.4%	1.00
	Right	71, 55.5%	239, 49.3%		21, 51.2%	99, 44.6%		13, 40.6%	52, 42.6%	
	Bilateral	0, 0%	8, 1.6%		2, 4.9%	5, 2.2%		0, 0%	0, 0%	
Distribution quadrant of lesions	Upper left	25, 19.5%	156, 32.2%	0.24	11, 26.8%	69, 31.1%	0.41	12, 37.5%	40, 32.8%	0.72
	Lower left	16, 12.5%	46, 9.5%		1, 2.4%	32, 14.4%		6, 18.8%	14, 11.5%	
	Upper right	43, 33.6%	153, 31.5%		10, 24.4%	54, 24.3%		8, 25%	42, 34.4%	
	Lower right	16, 12.5%	46, 9.5%		7, 17.1%	25, 11.3%		3, 9.4%	6, 4.9%	
	Right areola and central area	12, 9.4%	37, 7.6%		5, 12.2%	20, 9%		1, 3.1%	4, 3.3%	
	Left areola and central area	16, 12.5%	47, 9.7%		7, 17.1%	22, 9.9%		2, 6.2%	16, 13.1%	
Signal on T2WI	Hyperintensity	16, 12.5%	65, 13.4%	0.75	0, 0%	16, 7.2%	0.44	3, 9.4%	5, 4.1%	0.34
	Slightly high signal	101, 78.9%	364, 75.06%		34, 82.9%	177, 79.7%		24, 75%	105, 86.1%	
	Isointensity	8, 6.3%	45, 9.27%		7, 17.1%	24, 10.8%		2, 6.2%	9, 7.4%	
	Hypointensity	3, 2.3%	11, 2.27%		0, 0%	5, 2.3%		3, 9.4%	3, 2.4%	
Intratumoral edema	Present	52, 40.6%	137, 28.2%	**0.02**	10, 24.4%	63, 28.4%	0.98	12, 37.5%	43, 35.2%	1.0
	Absent	76, 59.4%	348, 71.8%		31, 75.6%	159, 71.6%		20, 62.5%	79, 64.8%	
Peritumoral edema	Present	64, 50%	159, 32.8%	**0.002**	20, 48.8%	74, 33.3%	0.28	17, 53.1%	48, 39.3%	0.41
	Absent	64, 50%	326, 67.2%		21, 51.2%	148, 66.7%		15, 46.9%	74, 60.7%	
Lesion enhancement type	Mass	91, 71.1%	423, 87.2%	**<0.001**	28, 68.3%	188, 84.7%	0.11	22, 68.8%	111, 91%	**0.05**
	Mass with NME	37, 28.9%	62, 12.8%		13, 31.7%	34, 15.3%		10, 31.2%	11, 9%	
Mass shape	Round or oval	43, 33.6%	138, 28.5%	0.46	11, 26.8%	65, 29.3%	1.0	4, 12.5%	19, 15.6%	0.94
	Irregular	85, 66.4%	347, 71.5%		30, 73.2%	157, 70.7%		28, 87.5%	103, 84.4%	
Mass margin	Circumscribed	1, 0.8%	12, 2.5%	0.46	0, 0%	4, 1.8%	1.00	1, 3.1%	8, 6.6%	0.86
	Irregular or spiculated	127, 99.2%	473, 97.5%		41, 100%	218, 98.2%		31, 96.9%	114, 93.4%	
Mass internal enhancement	Homogeneous	1, 0.8%	16, 3.3%	0.36	1, 2.4%	9, 4.1%	0.98	0.77	2, 1.6%	0.73
	Heterogeneous	123, 96.1%	445, 91.8%		38, 92.7%	206, 92.8%		31, 96.9%	120, 98.4%	
	Rim		24, 4.9%		2, 4.9%	7, 3.2%				
EER		0.62 (0.52, 0.7)	0.64 (0.54, 0.73)	0.37	0.6 (0.53, 0.7)	0.64 (0.55, 0.75)	0.36	0.6 ± 0.14	0.63 ± 0.12	0.29
TIC	Wash-in	1, 0.78%	7, 1.44%	0.67	0, 0%	3, 1.4%	0.99	1, 3.1%	2, 1.6%	0.27
	Plateau	29, 22.66%	132, 27.22%		10, 24.4%	62, 27.9%		5, 15.6%	41, 33.6%	
	Wash-out	98, 76.56%	346, 71.34%		31, 75.6%	157, 70.7%		26, 81.3%	79, 64.8%	
ADC_min_ (x10^−3^ mm^2^/s)		1 (0.89, 1.11)	0.96 (0.85, 1.09)	0.09	1 (0.92, 1.1)	0.96 (0.88, 1.11)	0.66	0.99 ± 0.11	1.02 ± 0.21	0.41
Increased vascularity or AVS	Positive	62, 48.4%	160, 33%	**0.005**	19, 46.3%	76, 34.2%	0.44	23, 71.9%	56, 45.9%	0.08
	Negative	66, 51.6%	325, 67%		22, 53.7%	146, 65.8%		9, 28.1%	66, 54.1%	
Lymphadenectasis (Axillary or internal mammary)	Yes	62, 48.4%	153, 31.5%	**0.002**	20, 48.8%	66, 29.7%	0.12	13, 40.6%	33, 27%	0.41
	No	66, 51.6%	332, 68.5%		21, 51.2%	156, 70.3%		19, 59.4%	89, 73%	
Accompanying signs	Yes	55, 43%	110, 22.7%	**<0.001**	14, 34.1%	62, 27.9%	0.84	8.25%	40, 32.8%	0.72
	No	73, 57%	375, 77.3%		27, 65.9%	160, 72.1%		24, 75%	82, 67.2%	
BI-RADS	4	55, 43.0%	219, 45.2%	0.75	17, 41.5%	96, 43.2%	1.00	14, 43.8%	62, 50.8%	0.79
	5	73, 57.0%	266, 54.8%		24, 58.5%	126, 56.8%		18, 56.2%	60, 49.2%	

MRI, magnetic resonance imaging; HER2, human epidermal growth factor receptor 2; HR, hormone receptor; ER, estrogen receptor; PR, progesterone receptor; IDC, invasive ductal carcinoma; DCIS, ductal carcinoma *in situ*; ILC, invasive lobular carcinoma; FGT, fibroglandular tissue component; BPE, background parenchymal enhancement; T2WI, T2-weighted images; NME, non-mass enhancement; EER, early enhancement ratio; TIC, time-intensity curve; ADC, apparent diffusion coefficient; AVS, adjacent vessel sign; BI-RADS, breast imaging reporting, and data system.

Accompanying signs include nipple inversion, skin retraction, and pectoralis muscle invasion.

The data were expressed as counts (percentage), mean ± SD, or median (interquartile range). P-values were adjusted using the False Discovery Rate (FDR) method for all univariate comparisons. Features with FDR-adjusted p-value ≤0.05 were considered statistically significant.

Bold values indicate a statistically significant difference with P≤0.05.

For patients with HER2-low from HER2-zero breast cancers, there were 494 lesions (369 HER2-low and 125 HER2-zero) in the training set, 213 lesions (166 HER2-low and 47 HER2-zero) in the internal validation, and 122 lesions (93 HER2-low and 29 HER2-zero) in the external validation set (Figure 1). Table 2 summarized the clinicopathological characteristics of HER2-low and HER2-zero breast cancer patients. In the training set, ER status (FDR-adjusted p-value = 0.02) and molecular subtype (FDR-adjusted p-value = 0.005) were statistically significant between HER2-low and HER2-zero breast cancer patients. The proportion of ER was higher in HER2-low breast cancer. In the internal validation set, the HER2-low and HER2-zero breast cancer patients were significantly different in HR status, ER status, PR status, and Ki-67 status (FDR-adjusted p-values <0.05). Additionally, among the molecular subtypes, triple-negative breast cancer was more prevalent in HER2-zero patients, whereas the Luminal subtype was more common in HER2-low patients. Significant differences were observed in these molecular subtypes across the training, internal, and external validation sets (FDR-adjusted p-values <0.05). However, patient age, menopausal status, histological grade, lymph node staging, and tumor staging did not demonstrate statistical significance (FDR-adjusted p-values >0.05).

**TABLE 2:** Clinicopathological and MRI features of HER2-low and HER2-zero breast cancer patients in the training, internal validation, and external validation sets.

Features		Training set (n = 494 lesions)		FDR-adjusted p-value	Internal validation set (n = 213 lesions)		FDR-adjusted p-value	External validation set (n = 122 lesions)		FDR-adjusted p-value
		HER2-low (n = 369 lesions)	HER2-0 (n = 125 lesions)		HER2-low (n = 166 lesions)	HER2-0 (n = 47 lesions)		HER2-low (n = 93 lesions)	HER2-0 (n = 29 lesions)	
Age		53.26 ± 11.62	53.71 ± 10.62	0.85	54 (47,63)	51 (45,55)	0.22	54.52 ± 9.92	54.83 ± 10.05	1.00
Menopausal status	Postmenopausal	198, 53.7%	71, 56.8%	0.84	93, 56%	20, 42.6%	0.35	62, 66.7%	20, 69%	1.00
	Premenopausal	171, 46.3%	54, 43.2%		73, 44%	27, 57.4%		31, 33.3%	9, 31%	
Tumor diameter (cm)		2.2 (1.6,3.1)	2 (1.6,2.7)	0.11	2 (1.6,2.7)	2.1 (1.65,2.7)	1.00	2 (1.5,2.6)	2 (1.3,2.4)	1.00
Histological grade	I	27, 7.3%	3, 2.4%	0.11	14, 8.4%	1, 2.1%	0.13	7, 7.5%	1, 3.45%	1.00
	II	239, 64.8%	77, 61.6%		111, 66.9%	26, 55.3%		57, 61.3%	19, 65.52%	
	III	103, 27.9%	45, 36%		41, 24.7%	20, 42.6%		29, 31.2%	9, 31.03%	
Regional N category	N0	206, 55.8%	81, 64.8%	0.48	100, 60.2%	29, 61.7%	1.0	56, 60.2%	17, 58.62%	0.75
	N1	140, 37.9%	38, 30.4%		60, 36.2%	18, 38.3%		33, 35.5%	9, 31.03%	
	N2	15, 4.1%	3, 2.4%		5, 3%	0, 0%		4, 4.3%	2, 6.9%	
	N3	8, 2.2%	3, 2.4%		1, 0.6%	0, 0%		0, 0%	1, 3.45%	
Primary T category	T1	158, 42.8%	65, 52%	0.25	82, 49.4%	22, 46.8%	0.84	48, 51.6%	17, 58.62%	0.73
	T2	187, 50.7%	56, 44.8%		77, 46.4%	25, 53.2%		41, 44.1%	9, 31.03%	
	T3	24, 6.5%	4, 3.2%		7, 4.2%	0, 0%		4, 4.3%	3, 10.35%	
HR status	Positive (ER and/or PR positive)	310, 84%	92, 73.6%	0.07	147, 88.6%	31, 66%	**0.007**	75, 80.6%	16, 55.2%	0.12
	Negative (ER and PR negative)	59, 16%	33, 26.4%		19, 11.4%	16, 34%		18, 19.4%	13, 44.8%	
ER status	Positive≥1%	306, 82.9%	88, 70.4%	**0.02**	144, 86.7%	30, 63.8%	**0.007**	73, 78.5%	16, 55.2%	0.19
	Negative<1%	63, 17.1%	37, 29.6%		22, 13.3%	17, 36.2%		20, 21.5%	13, 44.8%	
PR status	Positive≥1%	283, 76.7%	83, 66.4%	0.08	131, 78.9%	26, 55.3%	**0.01**	68, 73.1%	15, 51.7%	0.20
	Negative<1%	86, 23.3%	42, 33.6%		35, 21.1%	21, 44.7%		25, 26.9%	14, 48.3%	
Ki-67 status	Low (<20%)	135, 36.6%	31, 24.8%	0.08	66, 39.8%	5, 10.6%	**0.003**	40, 43%	6, 20.7%	0.20
	High (≥20%)	234, 63.4%	94, 75.2%		100, 60.2%	42, 89.4%		53, 57%	23, 79.3%	
Molecular subtype	Luminal A	134, 36.31%	29, 23.20%	**0.005**	68,40.96%	4, 8.51%	**<0.001**	36, 38.71%	5, 17.24%	**0.03**
	Luminal B	181, 49.05%	65, 52%		81,48.80%	26, 55.32%		39, 41.93%	12, 41.38%	
	HER2-enriched	0, 0	0, 0		0, 0	0, 0		0, 0	0, 0	
	Triple-negative	54, 14.64%	31, 24.8%		17, 10.24%	17, 36.17%		18, 19.36%	12, 41.38%	
Histological subtype	IDC	283, 76.7%	88, 70.4%	0.42	130, 78.31%	37, 78.7%	1.00	48, 51.6%	22, 75.9%	0.31
	IDC + DCIS	48, 13%	15, 12%		20, 12.05%	4, 8.5%		36, 38.7%	5, 17.2%	
	ILC	11, 3%	5, 4%		8, 4.82%	2, 4.3%		3, 3.2%	0, 0%	
	Mucinous	7, 1.9%	4, 3.2%		1, 0.6%	0, 0%		6, 6.5%	2, 6.9%	
	Other types	20, 5.4%	13, 10.4%		7, 4.22%	4, 8.5%		0, 0%	0, 0%	
FGT	Fatty or scattered	43, 11.7%	20, 16%	0.42	20, 12%	5, 10.6%	1.00	10, 10.8%	4, 13.8%	1.00
	Heterogeneous or extreme	326, 88.3%	105, 84%		146, 88%	42, 89.4%		83, 89.2%	25, 86.2%	
BPE	Minimal or mild	321, 87%	111, 88.8%	0.85	145, 87.3%	42, 89.4%	1.00	85, 91.4%	26, 89.7%	1.00
	Moderate or marked	48, 13%	14, 11.2%		21, 12.7%	5, 10.6%		8, 8.6%	3, 10.3%	
Lesion number	Single lesion	238, 64.5%	97, 77.6%	**0.05**	124, 74.7%	35, 74.5%	1.00	58, 62.4%	24, 82.8%	0.20
	Multifocal or multicentric lesions	131, 35.5%	28, 22.4%		42, 25.3%	12, 25.5%		35, 37.6%	5, 17.2%	
Lesion location	Left	188, 50.95%	64, 51.2%	1.00	84, 50.6%	20, 42.55%	0.77	54, 58.1%	16, 55.2%	1.00
	Right	175, 47.43%	59, 47.2%		79, 47.6%	25, 53.19%		39, 41.9%	13, 44.8%	
	Bilateral	6, 1.62%	2, 1.6%		3, 1.8%	2, 4.26%		0, 0%	0, 0%	
Distribution quadrant of lesions	Upper left	113, 30.6%	48, 38.4%	0.25	54, 32.53%	10, 21.28%	1.00	30, 32.26%	10, 34.48%	1.00
	Lower left	41, 11.1%	14, 11.2%		17, 10.24%	6, 12.77%		11, 11.83%	3, 10.34%	
	Upper right	103, 27.9%	36, 28.8%		53, 31.93%	15, 31.91%		30, 32.26%	12, 41.38%	
	Lower right	33, 9.0%	14, 11.2%		18, 10.84%	6, 12.77%		5, 5.38%	1, 3.45%	
	Right areola and central area	38, 10.3%	7, 5.6%		9, 5.42%	3, 6.38%		4, 4.3%	0, 0%	
	Left areola and central area	41, 11.1%	6, 4.8%		15, 9.04%	7, 14.89%		13, 13.97%	3, 10.35%	
Signal on T2WI	Hyperintensity	45, 12.2%	12, 9.6%	0.92	19, 11.45%	5, 10.64%	1.00	4, 4.3%	1, 3.4%	0.73
	Slightly high signal	278, 75.3%	97, 77.6%		130, 78.31%	36, 76.60%		77, 82.8%	28, 96.6%	
	Isointensity	35, 9.5%	13, 10.4%		16, 9.64%	5, 10.64%		9, 9.7%	0, 0%	
	Hypointensity	11, 3%	3, 2.4%		1, 0.6%	1, 2.12%		3, 3.2%	0, 0%	
Intratumoral edema	Present	106, 28.7%	38, 30.4%	0.92	41, 24.7%	15, 31.9%	0.84	32, 34.4%	11, 37.9%	1.00
	Absent	263, 71.3%	87, 69.6%		125, 75.3%	32, 68.1%		61, 65.6%	18, 62.1%	
Peritumoral edema	Present	117, 31.7%	43, 34.4%	0.85	58, 34.9%	15, 31.9%	1.00	42, 45.2%	6, 20.7%	0.19
	Absent	252, 68.3%	82, 65.6%		108, 65.1%	32, 68.1%		51, 54.8%	23, 79.3%	
Lesion enhancement type	Mass	316, 85.6%	114, 91.2%	0.26	141, 84.9%	40, 85.1%	1.00	85, 91.4%	26, 89.7%	1.00
	Mass with NME	53, 14.4%	11, 8.8%		25, 15.1%	7, 14.9%		8, 8.6%	3, 10.3%	
Mass shape	Round or oval	87, 23.6%	54, 43.2%	**<0.001**	43, 25.9%	19, 40.4%	0.22	16, 17.2%	3, 10.3%	1.00
	Irregular	282, 76.4%	71, 56.8%		123, 74.1%	28, 59.6%		77, 82.8%	26, 89.7%	
Mass margin	Circumscribed	4, 1.1%	6, 4.8%	0.08	4, 2.4%	2, 4.3%	1.00	6, 6.5%	2, 6.9%	1.00
	Irregular or spiculated	365, 98.9%	119, 95.2%		162, 97.6%	45, 95.7%		87, 93.5%	27, 93.1%	
Mass internal enhancement	Homogeneous	11, 2.98%	7, 5.6%	0.08	5, 3%	2, 4.26%	**0.03**	2, 2.2%	0, 0%	1.00
	Heterogeneous	346, 93.77%	108, 86.4%		158, 95.2%	39, 82.98%		91, 97.8%	29, 100%	
	Rim	12, 3.25%	10, 8%		3, 1.8%	6, 12.76%				
EER		0.65 (0.56, 0.75)	0.6 (0.5, 0.67)	**<0.001**	0.64 (0.57, 0.75)	0.62 (0.48, 0.7)	0.12	0.61 ± 0.16	0.52 ± 0.08	**0.002**
TIC	Wash-in	6, 1.6%	1, 0.8%	0.48	3, 1.8%	0, 0%	1.00	2, 2.2%	0, 0%	0.73
	Plateau	95, 25.8%	40, 32%		46, 27.7%	14,29.8%		28, 30.1%	13, 44.8%	
	Wash-out	268, 72.6%	84, 67.2%		117, 70.5%	33, 70.2%		63, 67.7%	16, 55.2%	
ADC_min_ (x10^−3^ mm^2^/s)		0.94 (0.85, 1.06)	1 (0.9, 1.12)	**0.01**	0.99 ± 0.21	1.1 ± 0.2	**0.01**	0.99 ± 0.21	1.14 ± 0.17	**0.002**
Increased vascularity or AVS	Positive	120, 32.5%	39, 31.2%	0.92	61, 36.7%	16, 34%	1.0	40, 43%	16, 55.2%	0.75
	Negative	249, 67.5%	86, 68.8%		105, 63.3%	31, 66%		53, 57%	13, 44.8%	
Lymphadenectasis (Axillary or internal mammary)	Yes	132, 35.8%	31, 24.8%	0.08	45, 27.1%	11, 23.4%	1.00	26, 28%	7, 24.1%	1.00
	No	237, 64.2%	94, 75.2%		121, 72.9%	36, 76.6%		67, 72%	22, 75.9%	
Accompanying signs	Yes	105, 28.5%	23, 18.4%	0.09	34, 20.5%	10, 21.3%	1.00	30, 32.3%	10, 34.5%	1.00
	No	264, 71.5%	102, 81.6%		132, 79.5%	37, 78.7%		63, 67.7%	19, 65.5%	
BI-RADS	4	161, 43.6%	53, 42.4%	0.92	72, 43.4%	29, 61.7%	0.13	46, 49.5%	16, 55.2%	1.00
	5	208, 56.4%	72, 57.6%		94, 56.6%	18, 38.3%		47, 50.5%	13, 44.8%	

MRI, magnetic resonance imaging; HER2, human epidermal growth factor receptor 2; HR, hormone receptor; ER, estrogen receptor; PR, progesterone receptor; IDC, invasive ductal carcinoma; DCIS, ductal carcinoma *in situ*; ILC, invasive lobular carcinoma; FGT, fibroglandular tissue component; BPE, background parenchymal enhancement; T2WI, T2-weighted images; NME, non-mass enhancement; EER, early enhancement ratio; TIC, time-intensity curve; ADC, apparent diffusion coefficient; AVS, adjacent vessel sign; BI-RADS, breast imaging reporting, and data system.

Accompanying signs include nipple inversion, skin retraction, and pectoralis muscle invasion.

The data were expressed as counts (percentage), mean ± SD, or median (interquartile range). P-values were adjusted using the False Discovery Rate (FDR) method for all univariate comparisons. Features with FDR-adjusted p-value ≤0.05 were considered statistically significant.

Bold values indicate a statistically significant difference with P≤0.05.

### 3.2 MRI features of HER2-positive, HER2-negative, HER2-low, and HER2-zero breast cancer patients

The MRI characteristics of patients with HER2-positive and HER2-negative breast cancer were compared. Supplementary Table S1 summarizes the inter-observer agreement for the MRI features. Agreement for MRI features was good to excellent (ICC, 0.88–0.96). Agreement for categorical MRI features was substantial to almost perfect (kappa, 0.74–0.88). As shown in Table 1, there was no significant difference in fibroglandular tissue composition, background parenchymal enhancement, lesion location, distribution quadrant, T2WI signal, mass shape, mass margin, mass internal enhancement pattern, EER, TIC, and BI-RADS classification between HER2-positive and HER2-negative patients in the training set (FDR-adjusted p-values >0.05). The tumor diameter for HER2-positive patients was 2.45 (1.9, 3) cm, significantly higher than that for HER2-negative patients (2.1 (1.6, 2.9) cm) (FDR-adjusted p-value = 0.02). Multifocal lesions, intratumoral and peritumoral edema, ipsilateral vascularity increase, lymphadenectasis, and other accompanying signs were more prominent in HER2-positive patients (FDR-adjusted p-values <0.05). Furthermore, masses with NME were observed in 28.9% of HER2-positive patients and 12.8% of HER2-negative patients, with significant differences (FDR-adjusted p-value <0.001). The median ADC value in the HER2-positive group was 1.00 (0.89, 1.11) × 10^−3^ mm^2^/s, which was comparable to that in the HER2-negative group (0.96 (0.85, 1.09) × 10^−3^ mm^2^/s; FDR-adjusted p-value = 0.09). In the internal validation set, multifocal or multicentric lesions were found in 53.9% of HER2-positive patients and 30.1% of HER2-negative patients, with this difference being statistically significant (FDR-adjusted p-value = 0.02), while no statistically significant differences were found in other MRI features (FDR-adjusted p-values >0.05). In the external validation set, differences emerged in the lesion enhancement type between HER2-positive and HER2-negative patients (FDR-adjusted p-value = 0.05). Two case examples involving HER2-positive patients are illustrated in Supplementary Figures S1 S2. The Supplementary Figure S1 depicts a case of HER2 positivity characterized by peritumoral edema and multiple heterogeneously enhancing oval masses. The Supplementary Figure S2 presents another HER2-positive case featuring masses with NME.

The MRI characteristics of patients with HER2-low and HER2-zero breast cancer are presented in Table 2. In the training set, the number of lesions, mass shape, EER, and ADC were found to be related to HER2 status. Specifically, patients with HER2-low breast cancer were more likely to exhibit multifocality, compared to those with HER2-zero status (FDR-adjusted p-value = 0.05). While HER2-low breast cancer predominantly exhibited irregular mass shapes, HER2-zero tumors could present as irregular, round, or oval, with proportions being similar between the two groups. The EER for HER2-low and HER2-zero breast cancers were 0.65 (0.56, 0.75) and 0.60 (0.50, 0.67), respectively, indicating statistical significance (FDR-adjusted p-value <0.001). The mean minimum ADC was significantly lower in HER2-low breast cancer (0.94 (0.85, 1.06) × 10^−3^ mm^2^/s) than in HER2-negative breast cancer (1.00 (0.90, 1.12) × 10^−3^ mm^2^/s) (FDR-adjusted p-value = 0.01). In the internal validation set, the internal enhancement patterns between the two groups demonstrated statistical significance (FDR-adjusted p-value = 0.03). The mean minimum ADC value for HER2-low breast cancer was (0.99 ± 0.21) × 10^−3^ mm^2^/s, significantly lower than that for HER2-negative breast cancer ((1.1 ± 0.2) × 10^−3^ mm^2^/s) (FDR-adjusted p-value = 0.01). In the external validation set, significant differences were found in EER (FDR-adjusted p-value = 0.002) and mean minimum ADC values (FDR-adjusted p-value = 0.002) between HER2-low and HER2-zero breast cancers. The imaging findings of two cases with HER2-low and HER2-negative status are presented in the Supplementary Figures S3, S4. Each had a single lesion characterized by mass enhancement. Supplementary Figure S3 depicts a case of HER2-low with an irregular tumor, an early enhancement rate of 0.84, and an ADC value of 0.83 × 10^−3^ mm^2^/s. Supplementary Figure S4 presents a case of HER2-negative status showing peritumoral edema and a rounded tumor, with an early enhancement rate of 0.58 and an ADC value of 0.99 × 10^−3^ mm^2^/s.

### 3.3 Construction and performance evaluation of the models for differentiating HER2-positive from HER2-negative breast cancer

**FIGURE 2:**
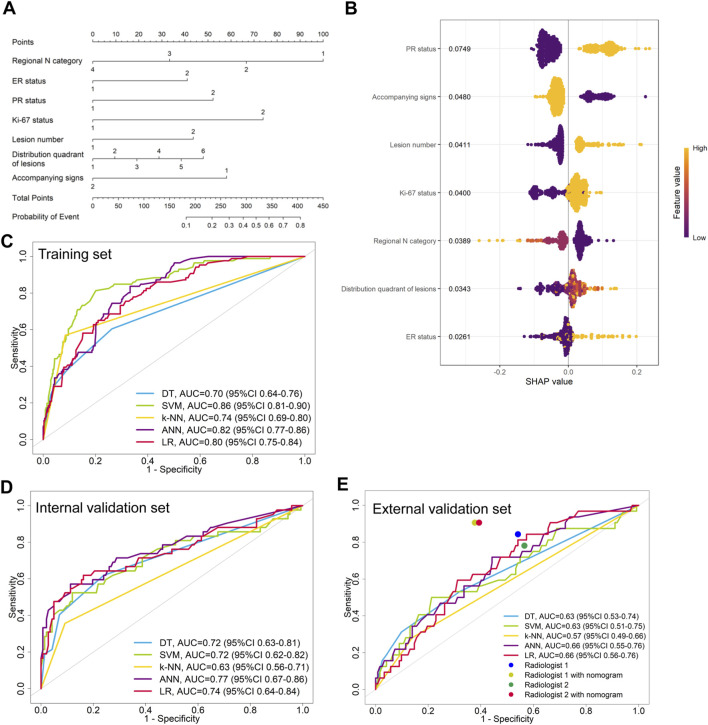
Model for differentiating HER2-positive from HER2-negative breast cancer. **(A)** Nomogram for predicting HER2-positive status, developed using the external validation set. **(B)** SHAP summary plot for the model differentiating HER2-positive from HER2-negative breast cancer. Each point represents a patient from the training set. The position on the x-axis shows the impact on the model output (SHAP value), and the color represents the feature value (yellow for high, purple for low). **(C–E)** ROC curves of the machine learning models in the **(C)** training, **(D)** internal validation, and **(E)** external validation sets.

**FIGURE 3:**
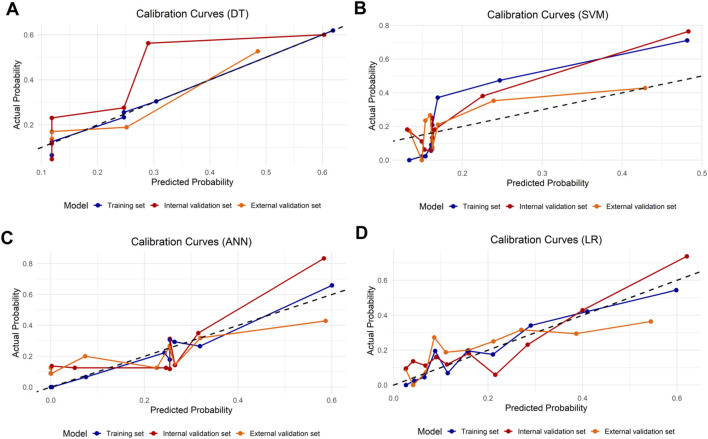
Model calibration for differentiating HER2-positive from HER2-negative breast cancer. Calibration curves for the **(A)** DT, **(B)** SVM, **(C)** ANN, and **(D)** LR models are shown for three sets. The dashed line represents ideal calibration.

**FIGURE 4:**
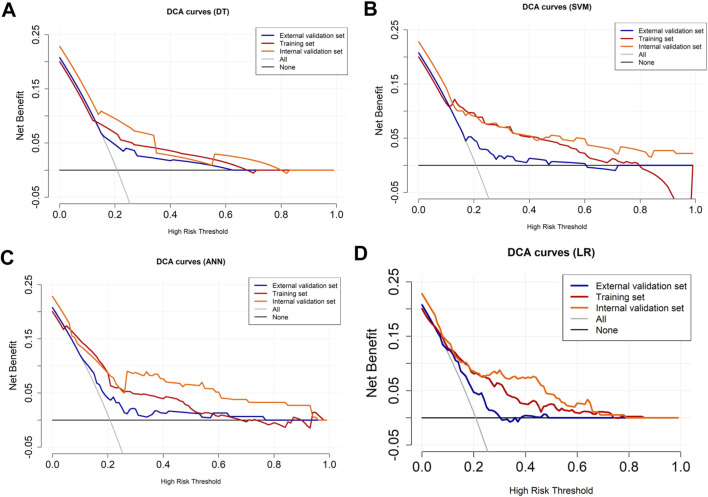
DCA curves for HER2-positive and HER2-negative breast cancer. The DCA curves of the **(A)** DT, **(B)** SVM, **(C)** ANN, and **(D)** LR models across all datasets. The black line represents the net benefit when no individuals receive the intervention (net benefit = 0), while the gray line represents the net benefit when all individuals receive the intervention.

LASSO regression identified the variables of clinical regional N category, ER status, PR status, Ki-67 status, lesion number, distribution quadrant of lesions, and accompanying signs. Then, multivariable logistic and stepwise regressions were used to construct the predictive models and the nomogram (Figure 2A). Furthermore, a SHAP analysis was conducted on the training datasets to assess the significance of each feature within the nomogram model. As shown in Figure 2B, PR status emerged as the most influential feature, with positive status substantially increasing the prediction probability for HER2-positive classification. Accompanying signs also demonstrated a notable impact, while ER status contributed least to the model’s output. The performances of the ML models to differentiate between HER2-positive and HER2-negative breast cancer were evaluated using ROC curves. In the training set, the AUC was 0.70 (95% confidence interval (CI): 0.64–0.76) for DT, 0.74 (95% CI: 0.69–0.80) for k-NN, 0.82 (95% CI: 0.77–0.86) for ANN, and 0.80 (95% CI: 0.75–0.84) for LR (Figure 2C; Table 3). The model of SVM had the highest AUC of 0.86 (95% CI: 0.81–0.90), with a sensitivity of 0.81 (95% CI: 0.70–0.90), a specificity of 0.80 (95% CI: 0.74–0.89), and an accuracy of 0.80 (95% CI: 0.76–0.86) (Table 3). In the internal validation set, the ANN model demonstrated highest discriminative ability, with an AUC of 0.77 (95% CI: 0.67–0.86), a sensitivity of 0.57 (95% CI: 0.43–0.83), a specificity of 0.89 (95% CI: 0.68–0.99), and an accuracy of 0.82 (95% CI: 0.69–0.88) (Figure 2D; Table 3). The AUC, sensitivity, specificity, and accuracy of the LR model were 0.74 (95% CI: 0.64–0.84), 0.62 (95% CI: 0.43–0.9079), 0.84 (95% CI: 0.77–0.97), and 0.79 (95% CI: 0.73–0.88), respectively (Figure 2D; Table 3). Additionally, the LR model achieved the highest AUC in the external validation set (0.66 (95% CI: 0.56–0.76)), a sensitivity of 0.59 (95% CI: 0.50–1.00), a specificity of 0.69 (95% CI: 0.25–0.79), and an accuracy of 0.67 (95% CI: 0.40–0.75) (Figure 2E; Table 3), although the overall performance was modest. The calibration curve for the models exhibited good agreement between the predicted risks and the observed probabilities across all three sets (Figure 3). The DCA (Figure 4) revealed that the net benefits of the various models in predicting HER2-positive and HER2-negative breast cancer across the three sets were high, indicating that the ML models have good clinical utility and practical application potential. The DCA-derived optimal threshold probabilities for clinical decision-making varied by model and dataset, ranging from 0.31 to 1.00 for this classification task (Table 3). Notably, the results of the k-NN model were classified as either 0 or 1, rather than representing probabilities of event occurrence; therefore, there was no DCA curve or calibration curve analysis for the k-NN model.

**TABLE 3:** Performance of 5 ML models in differentiating HER2-positive and HER2-negative breast cancer.

Model		The area under the curve (95% CI)	Sensitivity (95% CI)	Specificity (95% CI)	Accuracy (95% CI)	Negative predictive value (95% CI)	Positive predictive value (95% CI)	Optimal DCA threshold
Training set	DT	0.70 (0.64–0.76)	0.60 (0.35–0.71)	0.74 (0.70–0.94)	0.71 (0.68–0.83)	0.88 (0.85–0.91)	0.37 (0.32–0.63)	0.68
	SVM	0.86 (0.81–0.90))	0.81 (0.70–0.90)	0.80 (0.74–0.89)	0.80 (0.76–0.86)	0.94 (0.92–0.97)	0.50 (0.44–0.64)	0.80
	k-NN	0.74 (0.69–0.80)	0.57 (0.47–0.67)	0.92 (0.89–0.94)	0.85 (0.81–0.88)	0.89 (0.87–0.92)	0.63 (0.54–0.72)	*
	ANN	0.82 (0.77–0.86)	0.84 (0.70–0.99)	0.67 (0.48–0.79)	0.70 (0.58–0.79)	0.94 (0.91–0.99)	0.39 (0.32–0.48)	0.69
	LR	0.80 (0.75–0.84)	0.65 (0.57–0.91)	0.79 (0.56–0.87)	0.76 (0.62–0.82)	0.90 (0.89–0.96)	0.44 (0.33–0.56)	0.75
Internal validation set	DT	0.72 (0.63–0.81)	0.62 (0.36–0.76)	0.76 (0.70–0.96)	0.73 (0.67–0.85)	0.87 (0.82–0.91)	0.43 (0.37–0.77)	0.8
	SVM	0.72 (0.62–0.82)	0.52 (0.36–0.86)	0.88 (0.55–0.97)	0.80 (0.60–0.86)	0.86 (0.83–0.93)	0.56 (0.34–0.83)	1.00
	k-NN	0.63 (0.56–0.71)	0.36 (0.21–0.50)	0.91 (0.86–0.95)	0.78 (0.73–0.83)	0.83 (0.80–0.86)	0.54 (0.37–0.71)	*
	ANN	0.77 (0.67–0.86)	0.57 (0.43–0.83)	0.89 (0.68–0.99)	0.82 (0.69–0.88)	0.88 (0.84–0.94)	0.60 (0.40–0.89)	0.96
	LR	0.74 (0.64–0.84)	0.62 (0.43–0.79)	0.84 (0.77–0.97)	0.79 (0.73–0.88)	0.88 (0.84–0.93)	0.53 (0.44–0.84)	0.79
External validation set	DT	0.63 (0.53–0.74)	0.31 (0.16–0.69)	0.90 (0.63–0.98)	0.78 (0.62–0.83)	0.87 (0.83–0.91)	0.43 (0.36–0.76)	0.63
	SVM	0.63 (0.51–0.75)	0.50 (0.31–0.94)	0.79 (0.31–0.92)	0.73 (0.43–0.81)	0.86 (0.83–0.93)	0.56 (0.34–0.83)	0.61
	k-NN	0.57 (0.49–0.66)	0.28 (0.00–1.00)	0.86 (0.77–0.94)	0.74 (0.67–0.80)	0.83 (0.80–0.86)	0.54 (0.38–0.70)	*
	ANN	0.66 (0.55–0.76)	0.72 (0.34–0.97)	0.56 (0.26–0.91)	0.59 (0.40–0.81)	0.88 (0.84–0.93)	0.60 (0.40–0.89)	0.77
	LR	0.66 (0.56–0.76)	0.59 (0.50–1.00)	0.69 (0.25–0.79)	0.67 (0.40–0.75)	0.88 (0.84–0.92)	0.53 (0.44–0.84)	0.31
	Radiologist 1	0.62 (0.53–0.71)	0.84 (0.72–0.97)	0.46 (0.37–0.55)	0.54 (0.47–0.61)	0.92 (0.85–0.98)	0.29 (0.24–0.34)	--
	Radiologist 1 with nomogram	0.84 (0.76–0.91)	0.91 (0.56–1.00)	0.62 (0.57–0.94)	0.68 (0.64–0.89)	0.96 (0.89–1.00)	0.39 (0.35–0.77)	--
	Radiologist 2	0.58 (0.48–0.67)	0.78 (0.66–0.91)	0.43 (0.34–0.53)	0.51 (0.43–0.59)	0.88 (0.81–0.95)	0.27 (0.22–0.32)	--
	Radiologist 2 with nomogram	0.82 (0.74–0.89)	0.91 (0.56–1.00)	0.61 (0.54–0.93)	0.67 (0.61–0.87)	0.96 (0.89–1.00)	0.38 (0.34–0.68)	--

95% CI, 95% confidence interval; DT, decision tree; SVM, support vector machine; k-NN, k-nearest neighbors; ANN, neural network; LR, logistic regression; DCA, decision curve analysis. The optimal DCA, threshold represents the probability threshold that yielded the highest net benefit in the DCA, for each model in each dataset. *k-NN, model outputs class labels rather than probabilities; therefore DCA, threshold is not applicable. --, not applicable.

### 3.4 Construction and performance evaluation of the models for differentiating HER2-low from HER2-zero breast cancer

**FIGURE 5:**
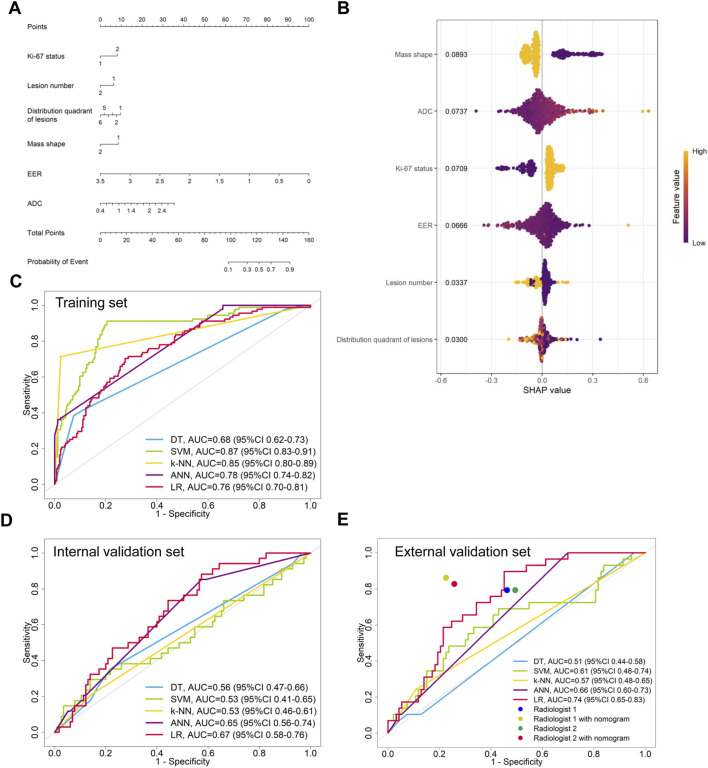
Model for differentiating HER2-low from HER2-zero breast cancer. **(A)** Nomogram for predicting HER2-low status, developed using the external validation set. **(B)** SHAP summary plot for the model differentiating HER2-low from HER2-zero breast cancer. The position on the x-axis shows the impact on the model output (SHAP value), and the color represents the feature value (yellow for high, purple for low). **(C–E)** ROC curves of the machine learning models in the **(C)** training, **(D)** internal validation, and **(E)** external validation sets.

**FIGURE 6:**
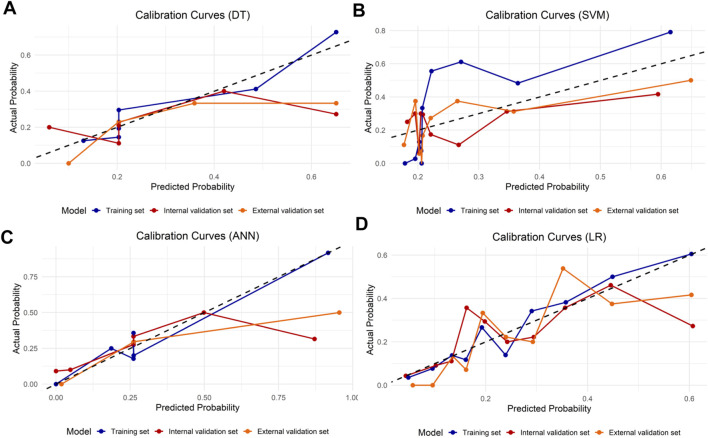
Calibration curves for HER2-low and HER2-zero breast cancer. Calibration curves of the **(A)** DT, **(B)** SVM, **(C)** ANN, and **(D)** LR models in three sets. The dashed line represents perfect calibration.

**FIGURE 7:**
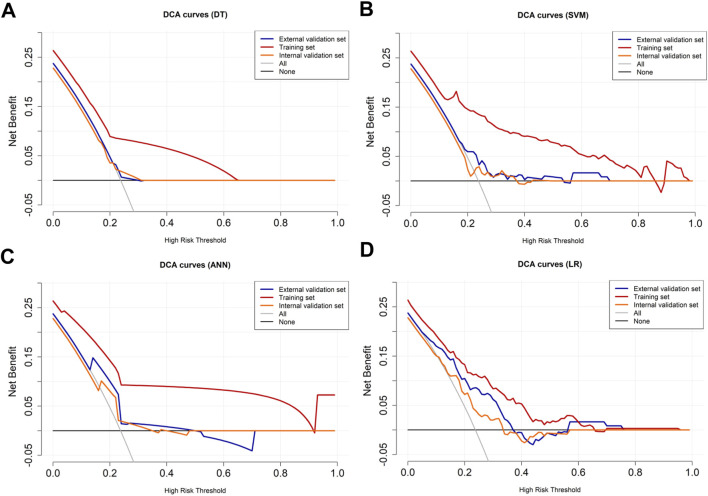
DCA curves for HER2-low and HER2-zero breast cancer. The DCA curves of the **(A)** DT, **(B)** SVM, **(C)** ANN, and **(D)** LR models across all datasets. The black line represents the net benefit when no individuals receive the intervention (net benefit = 0), while the gray line represents the net benefit when all individuals receive the intervention.

The variables of Ki-67 status, lesion number, distribution quadrant of lesions, mass shape, EER, and ADC were used for the construction of the predictive models and the nomogram (Figure 5A). SHAP analysis provided critical insights into the model’s decision-making process (Figure 5B). Mass shape emerged as the most influential feature, with irregular morphology substantially increasing the prediction probability for HER2-low classification. ADC values also demonstrated a significant impact, showing an inverse relationship with HER2-low probability, while the distribution quadrant of lesions contributed least to the model’s predictions. The performance of ML models in predicting HER2-low and HER2-zero breast cancer is summarized in Table 4 and Figures 5C–E. The SVM model achieved the highest AUC of 0.87 (95% CI: 0.83–0.91) in the training set, compared to DT with an AUC of 0.68 (95% CI: 0.62–0.73), k-NN with an AUC of 0.85 (95% CI: 0.80–0.89), ANN with an AUC of 0.78 (95% CI: 0.74–0.82), and LR with an AUC of 0.76 (95% CI: 0.70–0.81) (Figure 5C; Table 4). Conversely, the LR model achieved the highest AUCs in both the internal and external validation sets, with values of 0.67 (95% CI: 0.58–0.76) and 0.74 (95% CI: 0.65–0.83), respectively (Figures 5D,E; Table 4). The calibration curve (Figure 6) and DCA (Figure 7) illustrated that the ML models were well-calibrated and provided substantial clinical net benefits in predicting HER2-low and HER2-zero breast cancer across all three sets. The optimal threshold probabilities from the DCA for distinguishing HER2-low from HER2-zero tumors are presented in Table 4.

**TABLE 4:** Performance of 5 ML models in differentiating HER2-low and HER2-zero breast cancer.

Model		The area under the curve (95% CI)	Sensitivity (95% CI)	Specificity (95% CI)	Accuracy (95% CI)	Negative predictive value (95% CI)	Positive predictive value (95% CI)	Optimal DCA threshold
Training set	DT	0.68 (0.62–0.73)	0.38 (0.30–0.51)	0.93 (0.86–0.95)	0.78 (0.74–0.81)	0.81 (0.78–0.84)	0.65 (0.51–0.76)	0.66
	SVM	0.87 (0.83–0.91)	0.91 (0.85–0.97)	0.80 (0.74–0.85)	0.83 (0.79–0.87)	0.96 (0.94–0.99)	0.61 (0.56–0.69)	0.86
	k-NN	0.85 (0.80–0.89)	0.71 (0.63–0.80)	0.98 (0.96–0.99)	0.91 (0.88–0.93)	0.91 (0.88–0.93)	0.92 (0.85–0.97)	*
	ANN	0.78 (0.74–0.82)	0.36 (0.34–1.00)	0.99 (0.31–1.00)	0.82 (0.50–0.85)	0.81 (0.81–1.00)	0.92 (0.34–1.00)	0.92
	LR	0.76 (0.70–0.81)	0.70 (0.58–0.88)	0.72 (0.51–0.83)	0.72 (0.61–0.78)	0.87 (0.84–0.93)	0.48 (0.39–0.57)	0.66
Internal validation set	DT	0.56 (0.47–0.66)	0.35 (0.24–1.00)	0.78 (0.03–0.88)	0.68 (0.25–0.76)	0.80 (0.78–1.00)	0.32 (0.23–0.47)	0.32
	SVM	0.53 (0.41–0.65)	0.29 (0.12–0.91)	0.86 (0.24–0.98)	0.73 (0.39–0.81)	0.80 (0.78–0.91)	0.38 (0.25–0.77)	0.38
	k-NN	0.53 (0.46–0.61)	0.21 (0.00–1.00)	0.86 (0.00–1.00)	0.71 (0.23–0.77)	0.79 (0.77–0.82)	0.30 (0.23–0.50)	*
	ANN	0.65 (0.56–0.74)	0.85 (0.38–0.97)	0.43 (0.34–0.85)	0.52 (0.46–0.73)	0.91 (0.82–0.98)	0.31 (0.27–0.45)	0.35
	LR	0.67 (0.58–0.76)	0.88 (0.47–1.00)	0.43 (0.31–0.84)	0.53 (0.46–0.77)	0.92 (0.84–1.00)	0.31 (0.29–0.50)	0.34
External validation set	DT	0.51 (0.44–0.58)	1.00 (0.07–1.00)	0.04 (0.02–0.99)	0.27 (0.25–0.79)	0.80 (0.78–1.00)	0.32 (0.23–0.46)	0.3
	SVM	0.61 (0.49–0.74)	0.69 (0.31–0.86)	0.57 (0.48–0.90)	0.60 (0.54–0.79)	0.80 (0.78–0.91)	0.38 (0.25–0.75)	0.54
	k-NN	0.57 (0.48–0.65)	0.24 (0.00–1.00)	0.89 (0.00–1.00)	0.74 (0.24–0.80)	0.79 (0.77–0.82)	0.30 (0.23–0.50)	*
	ANN	0.66 (0.60–0.72)	1.00 (1.00–1.00)	0.30 (0.22–0.40)	0.47 (0.40–0.54)	0.91 (0.82–0.98)	0.31 (0.27–0.42)	0.5
	LR	0.74 (0.65–0.83)	0.90 (0.62–1.00)	0.55 (0.41–0.84)	0.63 (0.54–0.80)	0.92 (0.84–1.00)	0.31 (0.29–0.51)	0.38
	Radiologist 1	0.69 (0.58–0.79)	0.79 (0.34–0.93)	0.54 (0.44–0.90)	0.60 (0.52–0.79)	0.89 (0.81–0.96)	0.35 (0.29–0.54)	--
	Radiologist 1 with nomogram	0.81 (0.72–0.89)	0.86 (0.72–0.97)	0.77 (0.69–0.85)	0.80 (0.72–0.86)	0.95 (0.90–0.99)	0.54 (0.45–0.66)	--
	Radiologist 2	0.68 (0.58–0.78)	0.79 (0.31–0.93)	0.51 (0.42–0.90)	0.57 (0.51–0.80)	0.89 (0.80–0.96)	0.33 (0.29–0.59)	--
	Radiologist 2 with nomogram	0.77 (0.68–0.86)	0.83 (0.66–0.97)	0.74 (0.66–0.83)	0.76 (0.69–0.84)	0.93 (0.88–0.99)	0.50 (0.42–0.61)	--

95% CI, 95% confidence interval; DT, decision tree; SVM, support vector machine; k-NN, k-nearest neighbors; ANN, neural network; LR, logistic regression; DCA, decision curve analysis. The optimal DCA, threshold represents the probability threshold that yielded the highest net benefit in the DCA, for each model in each dataset. *k-NN, model outputs class labels rather than probabilities; therefore DCA, threshold is not applicable. --, not applicable.

### 3.5 Enhanced diagnostic performance of radiologists with nomogram models

In the external validation set, the LR models demonstrated the highest performance in differentiating between HER2-positive and HER2-negative breast cancer or between HER2-low and HER2-zero breast cancer. Then, nomogram models were constructed based on these models (Figures 2A, 5A). We compared the nomogram’s performance with that of two radiologists, finding that the nomogram outperformed both radiologists (Figure 2E; Table 3). The use of the nomogram enhanced the sensitivity, specificity, and accuracy of both radiologists (Table 3). In the evaluation of HER2-positive and HER2-negative breast cancer, the senior radiologist (Radiologist 1) exhibited improvements in the AUC, sensitivity, specificity, and accuracy by 0.22, 0.07, 0.16, and 0.14, respectively (Figure 2E; Table 3). For the junior radiologist (Radiologist 2), the enhancements in AUC, sensitivity, specificity, and accuracy were 0.24, 0.13, 0.18, and 0.14, respectively (Table 3; Figure 2E). In the analysis of HER2-low or HER2-zero breast cancer, the utilization of the nomogram led to an AUC, sensitivity, specificity, and accuracy of 0.77 (95% CI: 0.68–0.86), 0.83 (95% CI: 0.66–0.97), 0.74 (95% CI: 0.66–0.83), and 0.76 (95% CI: 0.69–0.84) for Radiologist 2, and 0.81 (95% CI: 0.72–0.89), 0.86 (95% CI: 0.72–0.97), 0.77 (95% CI: 0.69–0.85), and 0.80 (95% CI: 0.72–0.86) for Radiologist 1, respectively (Table 4; Figure 5E). Therefore, the integration of nomogram models significantly improved diagnostic accuracy in differentiating HER2-positive from HER2-negative and HER2-low from HER2-zero breast cancer, surpassing the performance of radiologists and highlighting the potential for enhanced clinical decision-making.

## 4 Discussion

Traditionally, HER2-low tumors were classified as HER2-negative, which led to missed opportunities for patients to receive anti-HER2 therapies (Marchio et al., 2021; Modi et al., 2022; Xin et al., 2022; Zhang et al., 2022; Yang et al., 2023). Current research indicates that nearly 50% of patients with HER2-negative breast cancer are classified as HER2-low, thereby enabling them to benefit from novel anti-HER2 treatments (Marchio et al., 2021; Modi et al., 2022; Xin et al., 2022; Zhang et al., 2022; Yang et al., 2023). Although preoperative biopsy can provide histological information, the biopsy samples may capture only focal aspects of the tumor and may not adequately represent the entire tumor compared to surgical samples (Chen et al., 2023). Preoperative breast MRI, however, provides comprehensive three-dimensional data, which is widely used to classify various histological types and molecular markers (Zhou et al., 2021; Ramtohul et al., 2023; Guo et al., 2024). This study investigated the value of clinical factors and imaging features extracted from preoperative MRI examinations for predicting the HER2 expression levels in breast cancer patients. The results demonstrated that clinicopathological and MRI features may serve as independent predictors for differentiating between HER2-positive and HER2-negative tumors, as well as for distinguishing HER2-low from HER2-zero tumors.

Our results demonstrated remarkable performance across the training, internal, and external validation sets in predicting HER2 status. Several clinicopathological variables, namely histologic grade, ER status, PR status, HR status, and Ki-67 index among HER2-positive, HER2-low, and HER2-zero breast cancer patients, were significantly different. ER-negative and PR-negative statuses were more frequently observed in HER2-positive patients compared to those with HER2-negative breast cancer, alongside higher histological grades and increased Ki-67 levels. Higher HR, ER, and PR statuses were noted in HER2-low tumors compared to HER2-zero cancers, associated with lower histological grades and lower Ki-67 levels, which is consistent with previous reports (Won et al., 2022; Chen et al., 2024). This may be attributed to the role of HER2 as a proto-oncogene that inhibits apoptosis and promotes proliferation. Such events are closely associated with various biological behaviors, including tumor cell invasion and metastasis (Zhu et al., 2021). Moreover, we found that compared to HER2-negative cancers, HER2-positive cancers were larger and exhibited more aggressive imaging features, including multifocal lesions, intratumoral and peritumoral edema, ipsilateral vascularity increase, lymphadenectasis, and other accompanying signs, which is consistent with the results of Zhou et al. (2025). The proportions of tumors with NME in HER2-positive and HER2-negative breast cancer patients were 28.9% and 12.8%, respectively. This difference is primarily associated with the greater presence of intraductal components in HER2-positive breast cancer (Seyfettin et al., 2022). Patients with HER2-low breast cancer were more likely to present multifocality, irregular shape compared to patients with HER2-zero tumors. Significant statistical differences were observed in the EER and mean minimum ADC values between HER2-low and HER2-zero breast cancers. These results are consistent with our previous findings (Zhao et al., 2024).

In our study, multivariate analyses were conducted to select clinicopathological and MRI features for the development of ML models. For distinguishing HER2-negative from HER2-positive tumors, the SVM model achieved the highest AUC of 0.86 (95% CI: 0.81–0.90) in the training set, while the LR model recorded AUCs of 0.74 (95% CI: 0.64–0.84) and 0.66 (95% CI: 0.56–0.76) in the internal and external validation sets, respectively. It is noteworthy that the observed decrease in performance in the external validation set is a common phenomenon in multicenter studies. Despite this expected attenuation, the model maintained statistically significant performance (AUC >0.5). These models showed good calibration and high net benefits in predicting HER2-positive and HER2-negative breast cancer across the three sets. These results are comparable to those of Zhou et al. (2025). Discrimination between HER2-low and HER2-zero cancers is critical, as lower agreement and accuracy among pathologists have been noted when interpreting scanned slides of HER2 1+ and HER2 0 scores in biopsies (Fernandez et al., 2022). In this study, the SVM model achieved the highest AUC of 0.87 (95% CI: 0.83–0.91) in the training set for differentiating HER2-low from HER2-zero tumors. Notably, the LR model outperformed all other models, including the ANN, yielding AUCs of 0.67 (95% CI: 0.58–0.76) and 0.74 (95% CI: 0.65–0.83) in the internal and external validation sets, respectively. These models demonstrated excellent calibration and provided significant clinical net benefits in predicting HER2-low and HER2-negative breast cancer across all three cohorts. Our results were comparable to predictions made by the radiomics model using breast MRI (Zhang et al., 2021; Xu et al., 2022; Zhou et al., 2025). The successful external validation, despite a slight performance drop, underscores the model’s practical generalizability and facilitates its potential application in diverse clinical settings. Overall, integrating ML-derived qualitative and quantitative features into the routine workflow as a supportive diagnostic tool will enhance our evaluation of HER2 status across the entire tumor.

Recently, imaging analysis driven by AI has emerged as a robust approach for extracting numerous quantitative characteristics from tumors. A standard breast MRI protocol involves the acquisition of various types of images, including T1-weighted imaging, T2WI, diffusion-weighted imaging, and DCE-MRI, which provide substantial information to train AI models for classification tasks. The predominant technique is radiomics analysis, enabling the direct extraction of features from the delineated tumors (Chen et al., 2020; Kim et al., 2021; Bian et al., 2023; Ramtohul et al., 2023; Zhang et al., 2023; Chen et al., 2024; Guo et al., 2024; Peng et al., 2024; Zheng et al., 2024). Several recent studies have established multiple models for identifying HER2 statuses using MRI findings or classic radiomics, achieving relatively strong performance (Bian et al., 2023; Ramtohul et al., 2023; Chen et al., 2024; Guo et al., 2024; Peng et al., 2024; Zheng et al., 2024). However, a significant limitation of radiomics models is their lack of interpretability, which hinders their clinical application (Chen et al., 2020; Kim et al., 2021; Bian et al., 2023; Ramtohul et al., 2023; Zhang et al., 2023; Chen et al., 2024; Guo et al., 2024; Peng et al., 2024; Zheng et al., 2024). Clinicians often find it challenging to understand how these models reach their conclusions and to identify which radiomic features are critical in the decision-making process. Our study directly addresses this “black-box” concern. By employing SHAP analysis, we have quantified and visualized the contribution of each feature, thereby making the model’s decision-making process transparent. Clinicians can not only obtain a prediction but also understand why that prediction was made—for instance, seeing that an irregular mass shape and a low ADC value were the key drivers for classifying a case as HER2-low. Additionally, radiomics models require specialized software, potentially limiting their utility in routine clinical practice (Chen et al., 2020; Kim et al., 2021; Bian et al., 2023; Ramtohul et al., 2023; Zhang et al., 2023; Chen et al., 2024; Guo et al., 2024; Peng et al., 2024; Zheng et al., 2024). In contrast to radiomics models, our study only collected clinicopathological data from the electronic medical record system and obtained radiologic imaging features directly from daily imaging workstations, without the need for complex three-dimensional tumor target delineation at various phases of enhanced scanning, which is computationally intensive. Notably, the clinicopathological and MRI features identified in our study, derived from a larger sample size and encompassing a more diverse set of features that are likely more representative, demonstrated comparable or slightly improved performance relative to established findings (Chen et al., 2020; Kim et al., 2021; Bian et al., 2023; Ramtohul et al., 2023; Zhang et al., 2023; Chen et al., 2024; Guo et al., 2024; Peng et al., 2024; Zhan et al., 2024; Zheng et al., 2024; Zhou et al., 2025).

Radiologists typically rely on morphological characteristics, such as tumor diameter, mass shape, mass margin, lesion enhancement type, enhancement rate, and ADC value, to diagnose breast lesions (Pinker et al., 2013). To our knowledge, this is the first study in which radiologists evaluated HER2 expression levels using breast MRI images. However, diagnostic accuracy often depends on the radiologist’s professional experience. The interpretation of breast MRI images can vary significantly among different observers, particularly among less experienced radiologists (Zhang et al., 2023). In the external validation set, we compared the predictive performance of the nomogram with the visual assessments made by radiologists. The nomogram demonstrated significantly higher sensitivity than that of both radiologists. By utilizing the nomogram, both sensitivity and specificity, as well as overall diagnostic accuracy, improved for the two radiologists, with more notable enhancements observed for the junior radiologist. This suggests that the high-dimensional features captured more nuanced information from breast MRI than could be identified by the naked eye. Therefore, the nomogram constructed in our study may significantly enhance the diagnostic capabilities of radiologists, particularly for those who are junior. The superior performance of the nomogram prompts the question of its decision-making rationale. Our SHAP analysis demystifies this process by quantifying the contribution of each feature, thereby building the trust necessary for clinical adoption. For discriminating HER2-positive from HER2-negative tumors, the model appropriately prioritized PR status, reflecting the well-established mutual inhibition between HER2 and hormone receptor pathways. Conversely, when identifying the clinically challenging HER2-low subtype, imaging phenotypes—specifically, mass shape and ADC values—surpassed receptor status in importance. This suggests that HER2-low tumors may exhibit distinct morphological and cellular characteristics discernible via MRI, providing a novel, non-invasive perspective for their identification.

Beyond diagnostic accuracy, we explored the potential for future clinical utility through a preliminary, data-driven estimation derived from our retrospective cohort. The results revealed that in our external validation, the use of the nomogram was associated with a reduction in the average interpretation time for a junior radiologist in our dataset. Furthermore, applying a conservative prediction threshold (probability of HER2-negative >0.95) identified a subset of patients for whom the model output a very low probability of positive or low HER2 expression. While these findings point to directions for future research into workflow efficiency and patient stratification, they remain speculative. The model’s performance and any potential impact on clinical workflows or decision-making must be rigorously tested in prospective studies before any clinical application can be considered.

This study has several limitations. First, it employed a retrospective design, which may introduce potential biases in patient selection. Second, the analysis was limited to three datasets derived from only two medical centers. Investigating a larger volume of prospectively collected data from additional centers could enhance the robustness of findings in future research. Third, a notable performance drop was observed in the external validation set. This is an expected phenomenon often attributed to domain shift, such as differences in MRI scanners, acquisition protocols, and patient populations across institutions (Park and Han, 2018; Kelly et al., 2019). While the successful external validation, despite this attenuation, underscores the model’s practical utility, the performance drop highlights the challenge of cross-institutional generalization. Future work should incorporate strategies such as post-acquisition image harmonization (e.g., ComBat) or domain adaptation techniques to improve model robustness. Fourth, the study excluded pure NME lesions. This decision was made because the standard BI-RADS features used in our model (e.g., mass shape, margin) are not directly applicable to NME, which is characterized by different imaging patterns. While this allowed us to develop a robust model for mass-forming tumors, it introduces a selection bias and limits the generalizability of our findings, particularly as NME is more common in HER2-positive breast cancers. Future studies are warranted to develop specific criteria or models for preoperatively predicting HER2 status in pure NME lesions, which would provide a more comprehensive clinical tool.

In conclusion, this study highlights the promising potential of integrating ML algorithms with clinicopathological and preoperative MRI characteristics for the classification of HER2 status in breast cancer. Our findings demonstrate that these models can effectively differentiate between HER2-positive, HER2-low, and HER2-zero tumors, potentially facilitating more informed therapeutic decisions. The nomogram models, specifically, significantly enhanced the diagnostic accuracy of radiologists, particularly benefiting those with less experience. Given the critical role of accurate HER2 classification in guiding therapeutic strategies, we advocate for the incorporation of these nomograms into routine clinical practice. Future multicentric studies with larger patient cohorts are necessary to further validate these models and ensure their robustness across diverse clinical settings.

## Data Availability

The raw data supporting the conclusions of this article will be made available by the authors, without undue reservation.
