# Supplementary material for: Enhancing preoperative HER2 status classification of invasive breast cancers using machine learning models based on clinicopathological and MRI features: a multicenter study
Source: Front Cell Dev Biol. 2025 Nov 26;13:1669651. doi: 10.3389/fcell.2025.1669651 (PMC12689904; doi:10.3389/fcell.2025.1669651)
Supplement: Supplementary file 1 [file DataSheet1.docx]

Supplementary Material

# Supplementary Figures


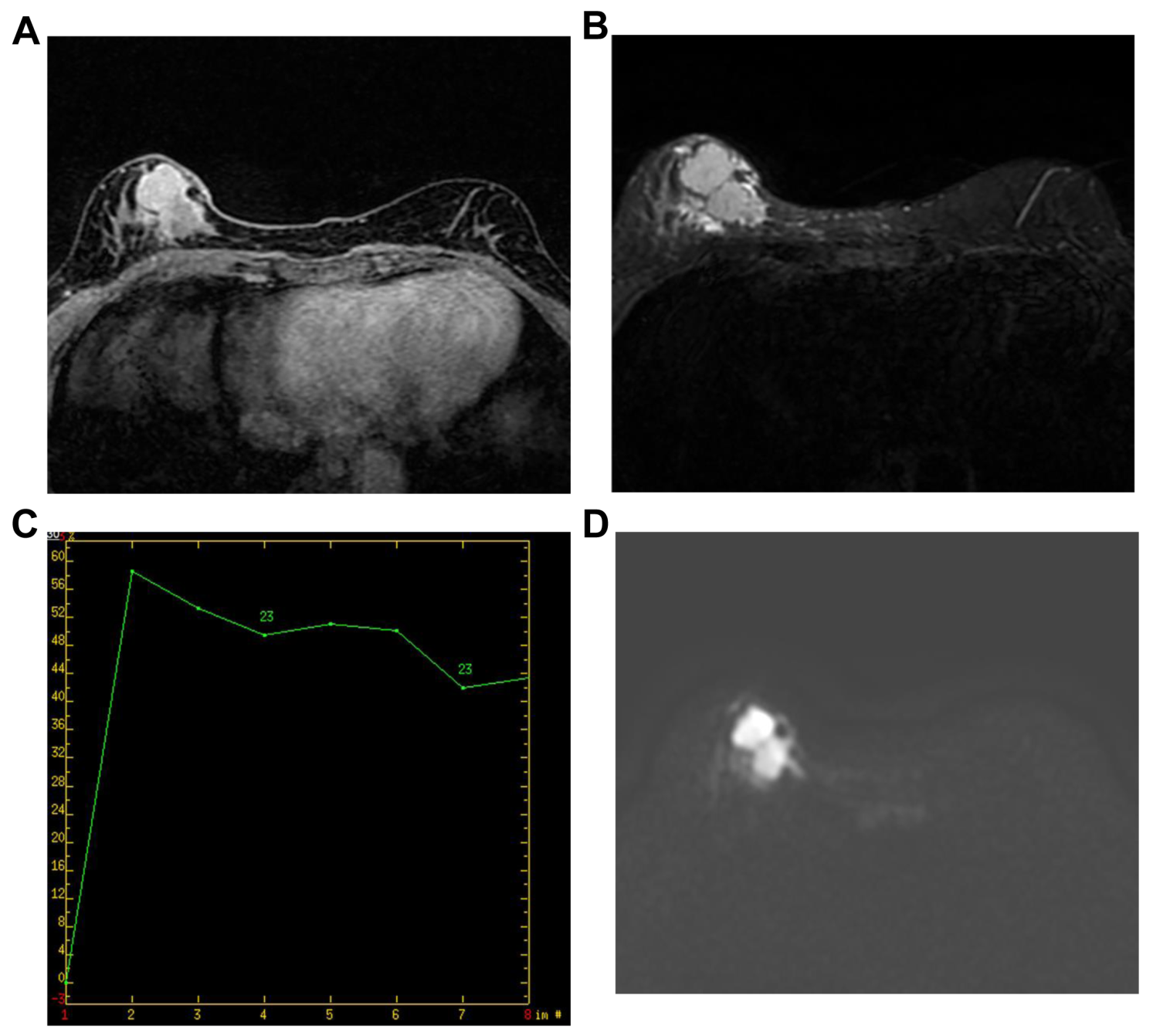


**Supplementary Figure S1. The MRI images of a 52-year-old female with invasive ductal carcinoma, HER2-positive, and histological grade III.** A. Contrast-enhanced axial T1-weighted MR image reveals multiple heterogeneously enhancing oval masses (largest: 2.4 cm) in the right inner-lower quadrant. B. Axial T2-weighted image demonstrates peritumoral edema (high signal). C. The time-intensity curve of the masses shows a type III (washout) kinetics with an early enhancement rate of 0.60. D. DWI shows a high signal with an ADC value of 0.91 × 10^-3^ mm^2^/s.


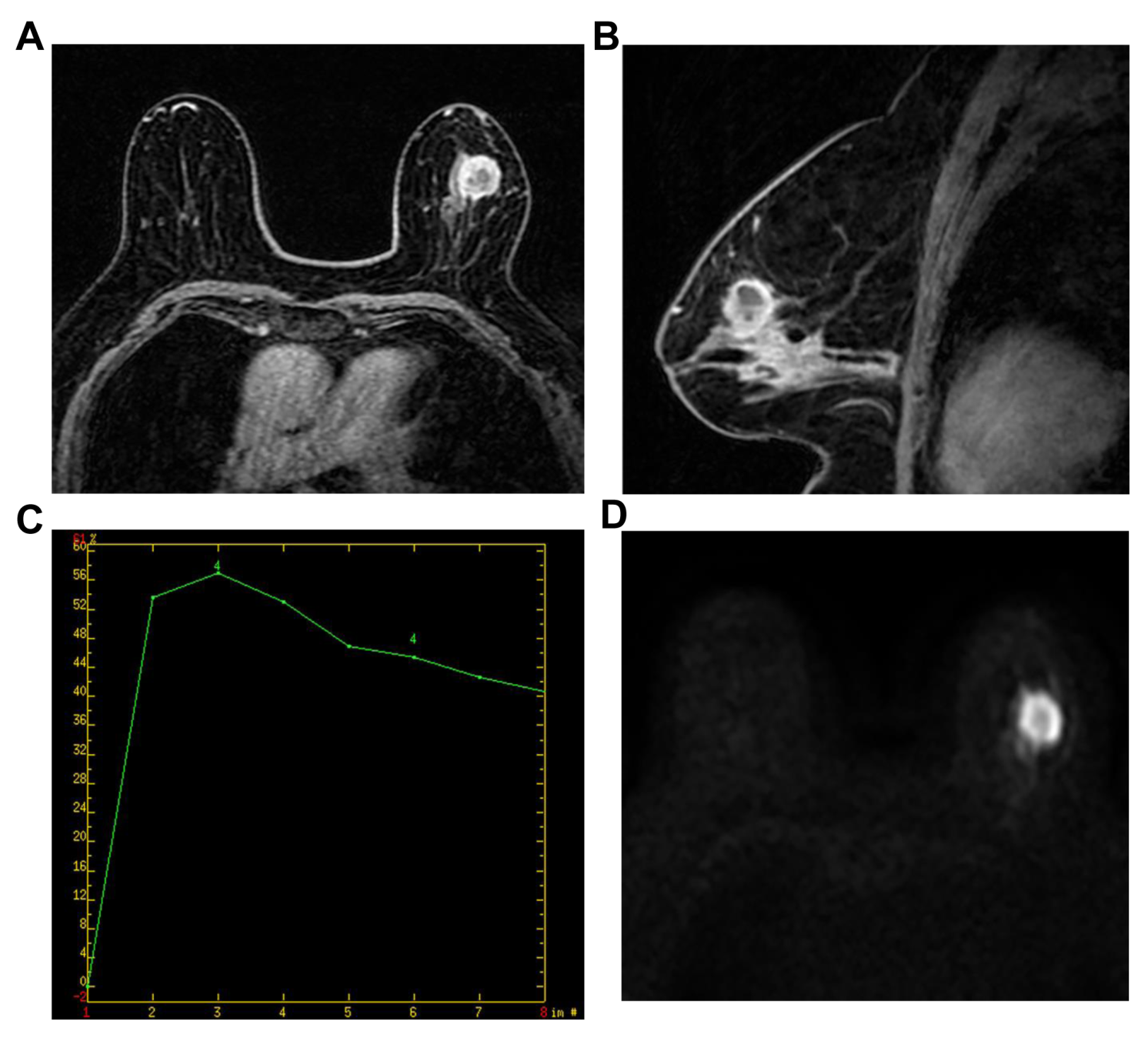


**Supplementary Figure S2. The MRI images of a 68-year-old female with invasive ductal carcinoma, HER2-positive, and histological grade III.** A. Contrast-enhanced axial T1-weighted image shows an annularly enhancing round mass (2.3 cm) in the left outer-upper quadrant. B. Contrast-enhanced sagittal T1-weighted image reveals adjacent non-mass enhancement surrounding the mass. C. The time-intensity curve shows type III (washout) kinetics with an early enhancement rate of 0.58. D. DWI shows a high signal with an ADC value of 0.99 × 10^-3^ mm^2^/s.


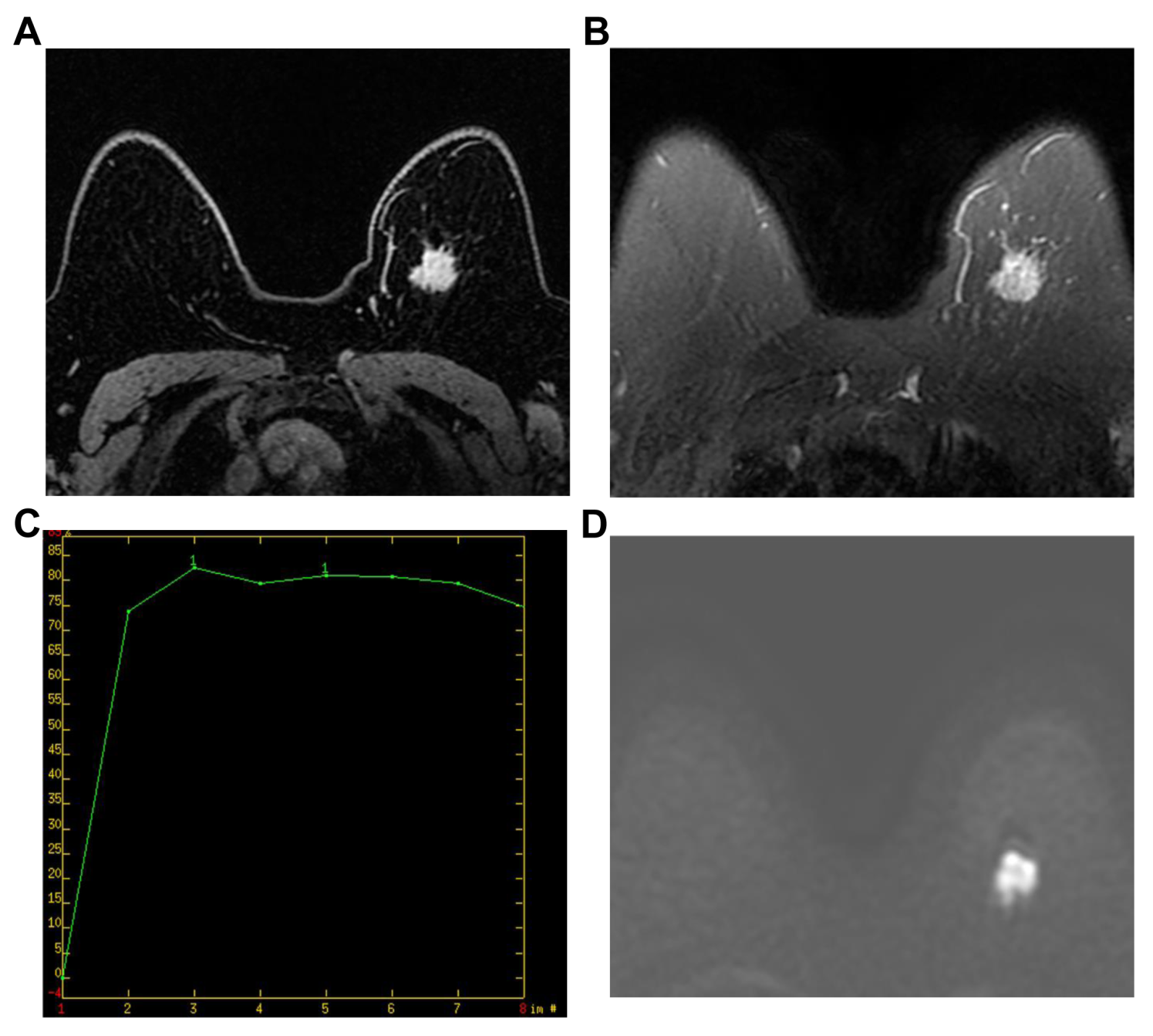


**Supplementary Figure S3.** **The MRI images of a 61-year-old female with invasive ductal carcinoma, HER2-low, and histological grade III.** A. Contrast-enhanced axial T1-weighted image shows a heterogeneously enhancing irregular mass (2.2 cm) in the left inner-upper quadrant. B. Axial T2-weighted image shows the mass with slightly high signal and no peritumoral edema. C. The time-intensity curve shows type II (plateau) kinetics with an early enhancement rate of 0.84. D. DWI shows a high signal with an ADC value of 0.83 × 10^-3^ mm^2^/s.


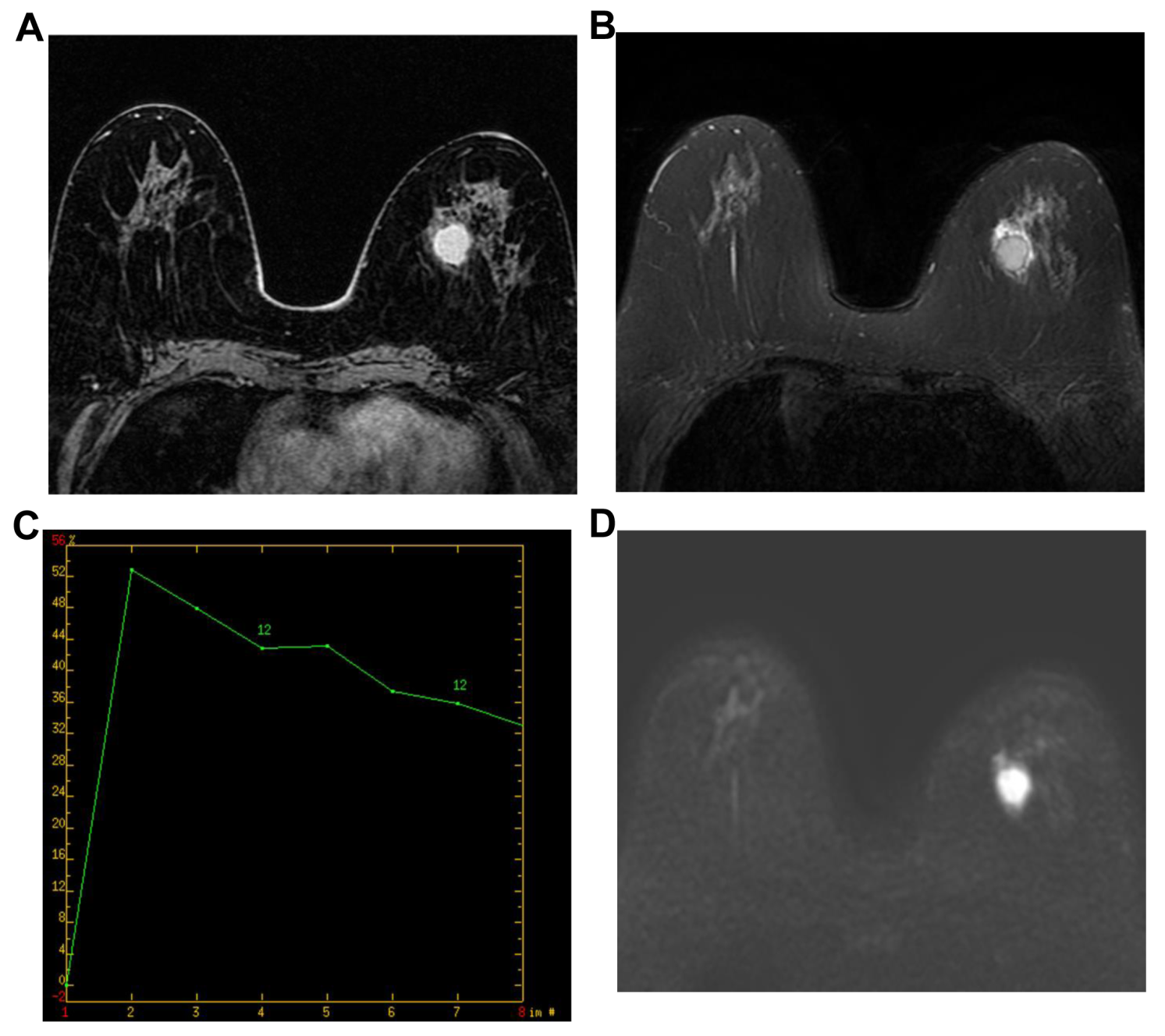


**Supplementary Figure S4. The MRI images of a 69-year-old female with invasive ductal carcinoma, HER2-negative, and histological grade III.** A. Contrast-enhanced axial T1-weighted image shows a heterogeneously enhancing round mass (2.2 cm) in the left inner breast. B. Axial T2-weighted image demonstrates peritumoral edema (high signal). C. The time-intensity curve shows type III (washout) kinetics with an early enhancement rate of 0.58. D. DWI shows high signal with a minimum ADC value of 0.99 × 10^-3^ mm^2^/s.

# Supplementary Table

# Supplementary Table S1. The inter-observer agreement for MRI features.

| **MRI features** | | **Agreement (95% confidence interval) *** |
| --- | --- | --- |
| Continuous variables | Tumor diameter | 0.91(0.82-0.98) |
|  | EER | 0.88 (0.84-0.93) |
|  | ADC | 0.96 (0.94-0.99) |
| Categorical variables | FGT | 0.80 (0.72-0.86) |
|  | BPE | 0.81 (0.78-0.84) |
|  | Multifocal or multicentric lesions | 0.79 (0.77-0.82) |
|  | Intratumoral edema | 0.78 (0.74-0.81) |
|  | Peritumoral edema | 0.76 (0.69-0.84) |
|  | Lesion enhancement type | 0.80 (0.78-0.91) |
|  | Mass shape | 0.80 (0.76-0.86) |
|  | Mass margin | 0.77 (0.68-0.86) |
|  | Mass internal enhancement | 0.83 (0.80-0.86) |
|  | TIC | 0.86 (0.83-0.93) |
|  | Lymphadenectasis | 0.74 (0.67-0.80) |
|  | Accompanying signs | 0.88 (0.81-0.95) |
|  | BI-RADS | 0.79 (0.73-0.88) |

Note: MRI, magnetic resonance imaging; FGT, fibroglandular tissue component; BPE, background parenchymal enhancement; EER, early enhancement ratio; TIC, time-intensity curve; ADC, apparent diffusion coefficient; BI-RADS, breast imaging reporting and data system.
 *Expressed as an intraclass correlation coefficient for continuous variables and as a kappa coefficient for categorical variables.
